# Trends and future directions of autophagy in osteosarcoma: A bibliometric analysis

**DOI:** 10.1515/med-2024-1080

**Published:** 2024-12-03

**Authors:** JinXiang Shang, FeiYing Zhao, Lu Xie, YaQing Wang, Bo Li, Cong Jin

**Affiliations:** Department of Orthopedics, Affiliated Hospital of Shaoxing University, Shaoxing, Zhejiang, China; Department of Sterilization and Supply Center, Zhuji People’s Hospital of Zhejiang Province, Shaoxing, Zhejiang, China; Department of Orthopedics, Beijing Luhe Hospital, Capital Medical University, Beijing, 100000, China; Department of Orthopedics, Shaoxing People’s Hospital, Shaoxing, 312000, Zhejiang, China

**Keywords:** autophagy, osteosarcoma, bibliometric analysis, invasion, cell death

## Abstract

**Background:**

Osteosarcoma, a highly malignant skeletal tumor, primarily affects children and adolescents. Autophagy plays a crucial role in osteosarcoma pathophysiology. This study utilizes bibliometric analysis to evaluate current research on autophagy in osteosarcoma and forecast future directions.

**Methods:**

We conducted a comprehensive search of publications in the Web of Science Core Collection database from January 1, 2008, to March 15, 2024. Tools like VOSviewer, CiteSpace, R software, Excel, and Scimago were used for analysis and visualization.

**Results:**

Publications increased steadily over 17 years, indicating rising interest. Zhang Yuan was the most influential author, with Shanghai Jiao Tong University leading. *Cell Death & Disease* was the top journal. “HMGB1 Promotes Drug Resistance in Osteosarcoma” was the most cited paper. Co-cited articles focused on drug resistance, therapeutic targets, autophagy in cancer, and genomic impacts on immunotherapy. Keywords highlighted invasion, migration, cell death, and breast cancer as research hotspots. Future studies will likely focus on therapeutic innovations and integrated management strategies.

**Conclusion:**

This bibliometric analysis offers an overview of current knowledge and emerging trends in autophagy and osteosarcoma, emphasizing key areas like invasion, migration, and cell death. It serves as a valuable resource for researchers developing novel therapies for osteosarcoma.

## 1 Introduction

Osteosarcoma, a severe form of malignancy affecting primarily the bones, disproportionately affects adolescents and young adults, leading to significant morbidity and mortality [1]. The disease’s complexity and the involvement of various cellular and molecular mechanisms pose significant challenges in developing effective treatment strategies [2]. Among the myriad factors contributing to osteosarcoma’s pathophysiology, autophagy has emerged as a pivotal element regulating cancer cell survival, proliferation, and therapy resistance [3].

Autophagy exhibits remarkable flexibility, allowing cells to adjust to various metabolic states and stress conditions in the tumor microenvironment [3]. Such adaptability has ignited significant interest in osteosarcoma research due to its impact on both the survival and destruction of cancer cells. While autophagy can support cellular survival in nutrient-scarce conditions, excessive activation may induce cell death. This balance is particularly pertinent in osteosarcoma, suggesting that modulation of autophagic pathways could unlock new therapeutic strategies [4]. The intricate balance between autophagy’s pro-survival and pro-death roles plays a pivotal part in osteosarcoma’s development, underlining the importance of autophagy as a target in the treatment of this cancer [5].

Within the tumor microenvironment of osteosarcoma, autophagy assumes a multifaceted role, shaped by diverse cellular stressors and environmental conditions [6,7]. This mechanism is vital for preserving cellular equilibrium under stress scenarios, including hypoxia and nutrient scarcity, prevalent in rapidly expanding tumors. Autophagy in osteosarcoma can enhance tumor cell survival by alleviating metabolic stress; yet, it may also lead to cell death when autophagic processes become overly active [8]. For the scope of this analysis, autophagy’s role within the tumor microenvironment is broadly addressed, with an understanding that its specific contributions might differ among various investigations. The modulation of autophagy in osteosarcoma cells plays a pivotal role in shaping the tumor’s therapeutic response and overall development. The delicate interplay between the protective and destructive facets of autophagy underscores its significance as a therapeutic target in osteosarcoma.

Deciphering the mechanisms regulating autophagy in osteosarcoma cells holds the key to unlocking their potential for tumor suppression, metabolic stress alleviation, and the induction of apoptosis [9]. Moreover, the creation of successful therapeutic strategies necessitates a profound comprehension of the complex interplay among cellular processes, various cell types within the tumor microenvironment, and the signaling molecules and pathways that enable their activation and intercommunication. Such a comprehensive understanding is crucial for devising innovative strategies that modulate autophagy in osteosarcoma, potentially paving the way for more effective treatments against this formidable cancer.

Considering the critical role of autophagy in osteosarcoma, it is crucial to assess the current research landscape in this area and identify knowledge gaps that require additional study. This bibliometric analysis aims to provide a comprehensive overview of the trends [10], key contributions, and leading figures and institutions that have markedly advanced our understanding of autophagy in the context of osteosarcoma. The goal is to guide future research directions and the development of new therapeutic approaches, thereby strengthening our fight against this cancer.

Bibliometric analysis acts as a pivotal tool in assessing the research landscape of a specific domain, offering insights into trends, critical contributions, and the key individuals, institutions, and countries involved [11]. Our goal, through a bibliometric analysis of autophagy in osteosarcoma, is to identify knowledge gaps, highlight emerging research areas, and outline promising future research directions. Furthermore, this method will illuminate the most influential publications, authors, and collaborations that have profoundly enhanced our comprehension of autophagy in osteosarcoma. In turn, this will guide future research efforts and encourage the innovation of new therapeutic strategies.

In this study, we present an extensive bibliometric analysis of the literature on autophagy in osteosarcoma. We identify pivotal publications, authors, institutions, and countries that have significantly influenced the field, while also exploring dominant research themes and trends. By mapping the knowledge landscape of autophagy research in osteosarcoma, we seek to provide a deeper insight into the field’s current state and encourage future research endeavors that could lead to innovative therapeutic approaches.

## 2 Materials and methods

### 2.1 Data screening and collection

The Web of Science Core Collection (WOSCC) database was employed for the bibliometric analysis, a common practice in this field. On March 15, 2024, we retrieved and downloaded literature from WOSCC spanning January 1, 2008, to March 15, 2024. Our search utilized terms such as “autophagy,” “autophagy, cellular,” “osteosarcoma,” and “osteosarcomas,” focusing specifically on articles and review articles in English. Two authors independently screened the results, excluding papers not relevant to both autophagy and osteosarcoma based on their titles, abstracts, and full texts. Discrepancies were resolved by a review from the senior corresponding author. Literature data were exported in the “full record and cited references” format and downloaded as plain text.

Citespace (Ver. 6.1.R6) and VOSviewer (Ver. 1.6.18) were used for the bibliometric analysis. Furthermore, Excel was utilized to illustrate the annual publication output concerning autophagy and osteosarcoma. The bibliometrix 4.1.3 tool within R software version 4.3.3 facilitated the conduct of Lotka’s Law analysis.

**Figure 1:**
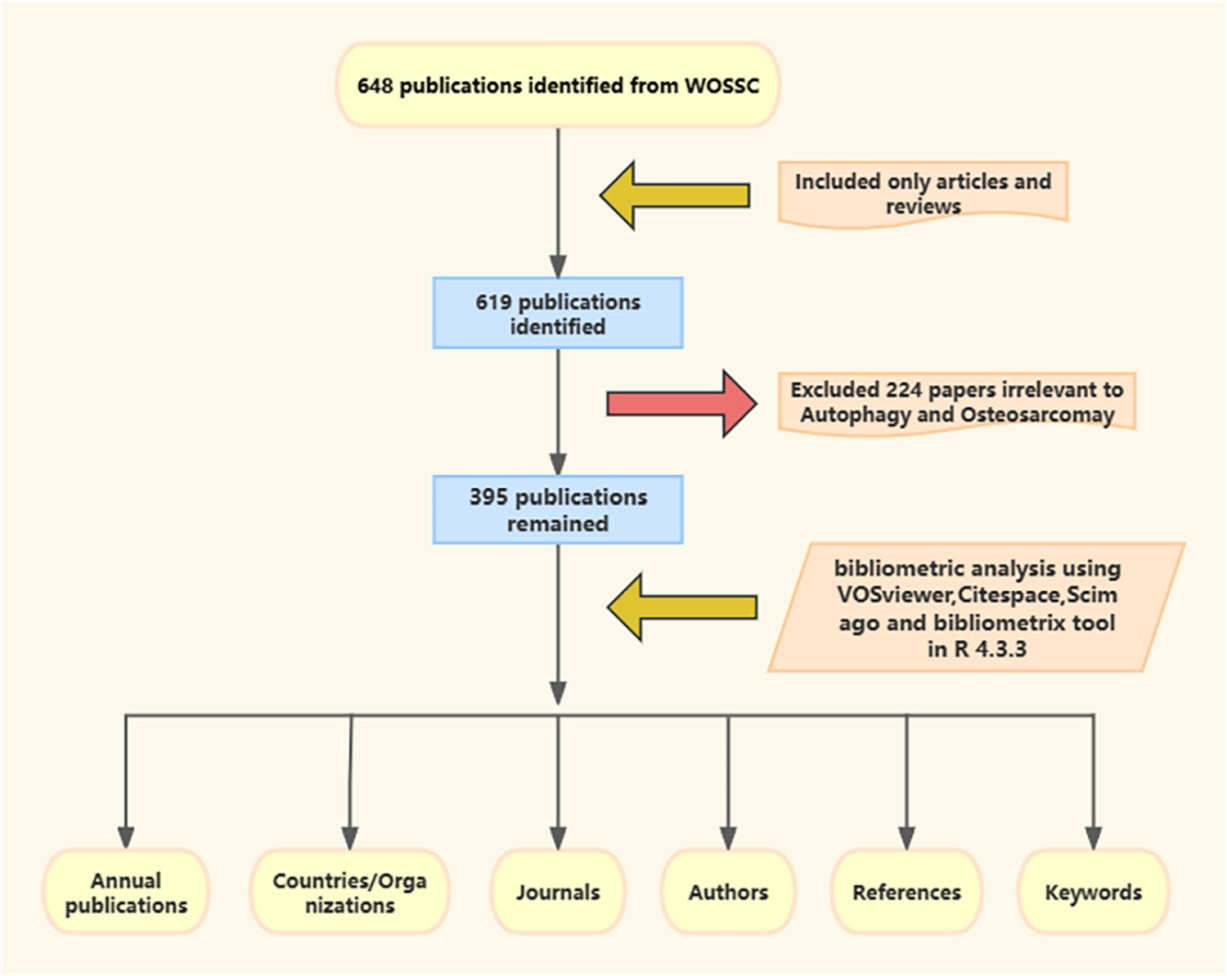
Publication screening flowchart.

VOSviewer, a complimentary Java-based software created by Van Eck and Walterman, facilitated the construction and generation of visual bibliometric maps [12]. This tool provides a range of straightforward visualizations, such as network, overlay, and density visualizations. Using VOSviewer, we developed co-authorship networks, performed citation analyses of countries, organizations, and authors, and created overlay visualization maps of references. Additionally, we generated density maps for co-authorship analysis of cited authors and conducted a co-occurrence analysis of all keywords. The flowchart of the data analysis process is illustrated in Figure 1.

### 2.2 Review method

An electronic literature search on the WOSCC identified peer-reviewed English articles related to autophagy and osteosarcoma, aiming to compile an expert narrative review. This search adhered to the same strategy as previously described. Initially, 648 documents were retrieved, but only those classified as “articles and reviews” were selected, narrowing the field to 619 documents. Of these, 224 did not fulfill the inclusion criteria. After evaluating content and removing irrelevant works, 395 articles were found relevant to autophagy and osteosarcoma and thus considered eligible (File S1). These articles were closely related to the topics of autophagy and osteosarcoma. The process of synthesizing bibliometric insights and incorporating articles pertinent to identified hotspots and frontiers resulted in 33 articles being selected for the narrative review, as illustrated in Table S1.

## 3 Results

### 3.1 Publication outputs and trends

**Figure 2:**
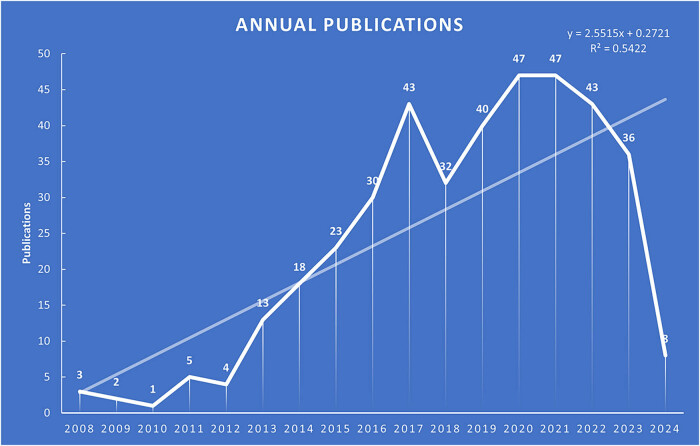
Annual output on autophagy in osteosarcoma.

Following our search criteria, we identified 395 papers on the interplay between autophagy and osteosarcoma for bibliometric analysis, spanning from January 1, 2008, to March 15, 2024. The annual publication frequency of this subject is depicted in Figure 2, showing a steady increase in publications over time. Between 2008 and 2012, publication numbers were relatively low, averaging fewer than six papers annually. However, from 2013 onwards, there was a marked increase in output. Specifically, from 2013 to 2023, the publication volume consistently exceeded 30 papers yearly (Figure 2). Microsoft Excel was used to construct publication trends for this topic, and the results suggested a high correlation between the number of annual publications and year (*y* = 2.5515*x* + 0.2721). Based on publication trends, it is predicted that 43 articles will be published on this topic by 2024, and the number of publications will reach 46 by 2025, indicating that an increasing number of scholars will focus on this area over time.

### 3.2 Countries and organizations

**Figure 3:**
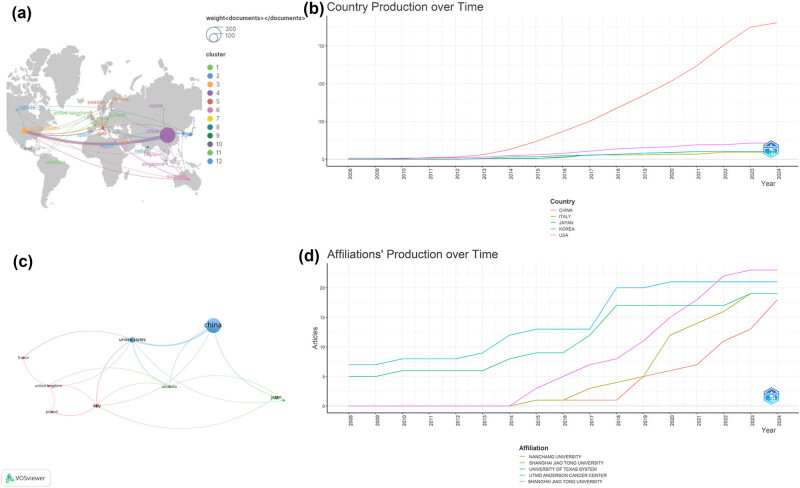
Visualization of country publications (a), country publication over time (b), co-authorship between countries (c), and affiliations’ publication over time (d) in research on autophagy in osteosarcoma.

Research publications in this field have emerged from 31 countries, involving 522 organizations. Leading the way, China produced 298 papers, accounting for 75.44% of total publications. It was followed by the United States with 43 papers (10.89%), Japan with 18 papers (4.56%), Italy with 16 papers (4.05%), and South Korea with 11 papers (2.78%) (Figure 3a). Over time, China’s publication volume has markedly increased, demonstrating a growth trend surpassing that of other countries (Figure 3b). However, among the leading 10 countries, the citation rate for China’s publications stands at a relatively lower average of 24.31 citations per paper. Conversely, France boasts the highest average citation rate at 45.57 citations, followed by the United States (34.58 citations), Japan (27.44 citations), South Korea (27.18 citations), Italy (23.38 citations), India (22.83 citations), and the United Kingdom (21.00 citations) (Table 1).

**Table 1:** The top 10 productive countries in the field of autophagy and osteosarcoma

Rank	Country	Documents	Percentage (%)	Total citations	Average citations	Percentage (%)
1	China	298	75.44	7.245	24.31	75.44
2	United States	43	10.89	1.487	34.58	10.89
3	Japan	18	4.56	494	27.44	4.56
4	Italy	16	4.05	374	23.38	4.05
5	South Korea	11	2.78	299	27.18	2.78
6	Australia	8	2.03	112	14.00	2.03
7	France	7	1.77	319	45.57	1.77
8	Poland	7	1.77	94	13.43	1.77
9	India	6	1.52	137	22.83	1.52
10	United Kingdom	5	1.27	105	21.00	1.27

International collaboration was evaluated through the analysis of country collaboration networks. In this network, each node symbolizes a country, and the node’s size generally reflects that country’s research activity within the dataset. The connections between nodes illustrate the collaborative relationships among countries, with the line thickness denoting the extent of collaboration. Here, thicker lines signify more frequent collaborations, whereas thinner lines suggest sporadic collaboration. Among the 31 participating countries, the United States and China exhibit the strongest and most frequent co-authorship (Figure 3c). Furthermore, the top 10 organizations, ranked by their publication count, contributed to 32.9% (130 out of 395) of the overall publications, with individual publication counts varying from 9 to 31 (Table 2).

**Table 2:** The top 10 productive organizations published literature related to autophagy and osteosarcoma

Rank	Organization	Country	Documents	Total citations	Average citations	Percentage (%)
1	Shanghai Jiao Tong Univ	China	31	1,043	33.65	7.85
2	Zhejiang Univ	China	19	751	39.53	4.81
3	Guangxi Med Univ	China	11	107	9.73	2.78
4	Wuhan Univ	China	10	173	17.30	2.53
5	Chongqing Med Univ	China	10	324	32.40	2.53
6	Shandong Univ	China	10	185	18.50	2.53
7	Nanjing Med Univ	China	10	257	25.70	2.53
8	Nanchang Univ	China	10	120	12.00	2.53
9	China Med Univ	China	10	250	25.00	2.53
10	Central South Univ	China	9	607	67.44	2.28

All top 10 organizations are located in China, with Shanghai Jiao Tong University at the forefront of publication volume, producing a total of 31 papers (7.85%) and garnering 1,043 citations. The publication output of this university has shown a consistent increase over time, indicating significant growth (Figure 3d). Zhejiang University ranks second, with 19 papers (4.81%) and 751 citations, followed by Guangxi Medical University with 11 papers (2.78%) and 107 citations. Remarkably, Central South University, despite publishing a smaller number of papers [9] related to autophagy and osteosarcoma from 2008 to 2024, boasts the highest average citation rate at 67.44 citations per paper (Table 2).

### 3.3 Journals and co-cited journals

**Figure 4:**
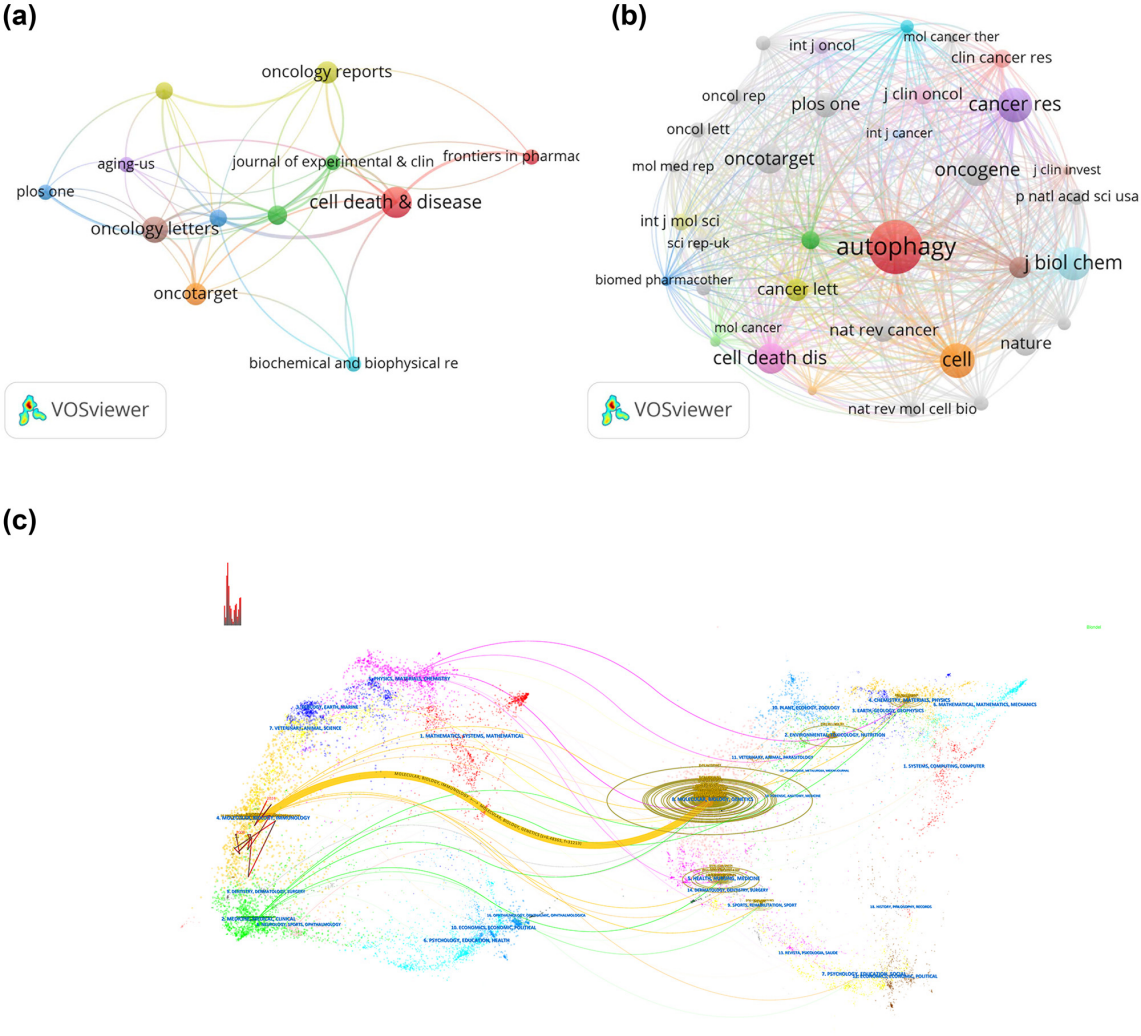
Visualization of journal publications (a) and cited journals (b) in research on autophagy in osteosarcoma, along with a dual-map overlay analysis of the citation relationships between journals (c).

Between 2008 and 2024, 184 academic journals published a total of 395 papers on the topic of autophagy and osteosarcoma. The top 10 journals accounted for 23.3% of all publications, as detailed in Table 3. *Cell Death & Disease* was the most prolific, publishing 14 papers (3.54% of the total), followed closely by *Oncology Letters* with 12 papers (3.04%), and both *Oncotarget* and *Oncology Reports*, each contributing 10 papers (2.53%). The *International Journal of Molecular Sciences* also made a notable contribution with 9 papers (2.28%) (Figure 4a).

**Table 3:** The most cited journals associated with autophagy and osteosarcoma

Rank	Journal	Count	Percentage (%)	Total citations	IF (2022)	JCR division (2022)
1	*Cell Death & Disease*	14	3.54	1010	9	Q1
2	*Oncology Letters*	12	3.04	375	2.9	Q3
3	*Oncotarget*	10	2.53	361	—	—
4	*Oncology Reports*	10	2.53	314	4.2	Q3
5	*International Journal of Molecular Sciences*	9	2.28	79	5.6	Q1
6	*International Journal of Oncology*	8	2.03	216	5.2	Q2
7	*Molecular Medicine Reports*	8	2.03	219	3.4	Q3
8	*Journal of Experimental & Clinical Cancer Research*	7	1.77	465	11.3	Q1
9	*PLoS One*	7	1.77	172	3.7	Q2
10	*Biochemical and Biophysical Research Communications*	7	1.77	200	3.1	Q3

In terms of total citations, the leading three journals were *Cell Death & Disease* with 1,010 citations, *Journal of Experimental & Clinical Cancer Research* with 465 citations, and *Oncology Letters* with 375 citations. Among the top 10 journals, 40% (4 out of 10) boasted an impact factor (IF) exceeding 5. From the 2,090 journals referenced, 35 received more than 100 citations each (Figure 4b). *Autophagy* leads in total citations with 577, followed by *Cancer Research* with 373, *Cell* with 368, and *Journal of Biological Chemistry* with 356 citations. Within the top 10 co-cited journals, 70% (7 out of 10) have an IF above 5, and the same proportion (70%) are ranked in the JCR Q1 zone (Table 4).

**Table 4:** The most co-cited journals associated with autophagy and osteosarcoma

Rank	Co-cited journal	Total citations	IF (2022)	JCR division (2022)
1	*Autophagy*	577	13.3	Q1
2	*Cancer Research*	373	11.2	Q1
3	*Cell*	368	64.5	Q1
4	*Journal of Biological Chemistry*	356	4.8	Q2
5	*Oncogene*	348	8.0	Q1
6	*Cell Death & Disease*	325	9.0	Q1
7	*Oncotarget*	313	—	—
8	*PLoS One*	269	3.7	Q2
9	*Nature*	258	64.8	Q1
10	*Cancer Letters*	239	9.7	Q1

Regarding changes in trends of research disciplines, we employed a dual-map overlay analysis to visualize the citation relationship between journals and reveal interdisciplinary crossovers. The left side of Figure 4c displays a basic graph of the citing journals, while the right side shows the cited journals. In Figure 4c, the thickest stripe represents the core citation path. The orange path indicates that articles published in molecular/biological/immunology journals on autophagy and osteosarcoma typically cite molecular/biological/genetics journals.

### 3.4 Authors and co-cited authors

**Figure 5:**
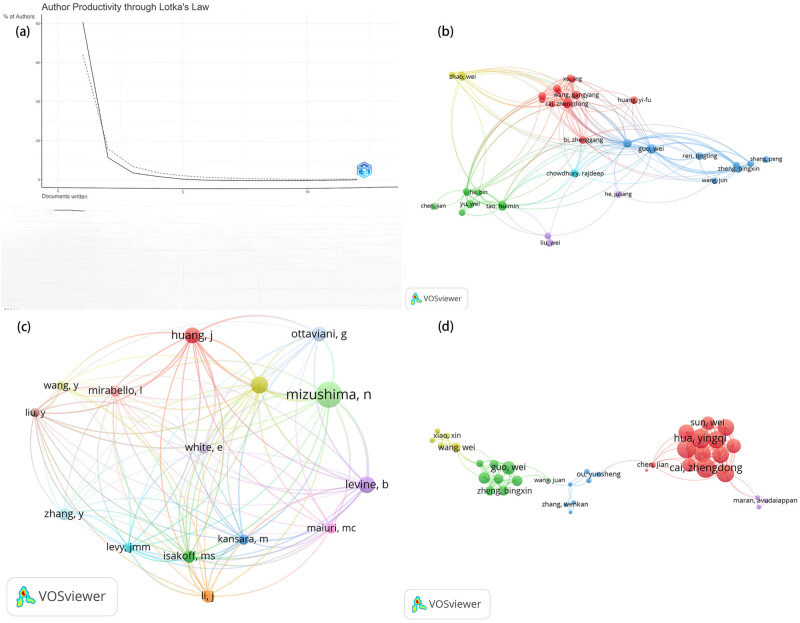
Analysis of authors’ publications according to Lotka’s law (a), visualization of authors (b), co-cited authors (c), and co-authorship authors (d) in research on autophagy in osteosarcoma.

In the study of autophagy and osteosarcoma, a total of 2,118 authors have contributed to the research. An application of Lotka’s law to assess scientific productivity reveals that a predominant 80.70% of these authors published only a single paper. Additionally, 11.60% published two papers, and a smaller fraction, 3.80%, published three papers, as shown in Figure 5a. Figure 5b identifies 39 authors who have contributed four or more papers in this domain. In terms of co-citations, 11,240 authors were mentioned at least once, with 15 authors receiving 35 or more co-citations, depicted in Figure 5c.

**Figure 6:**
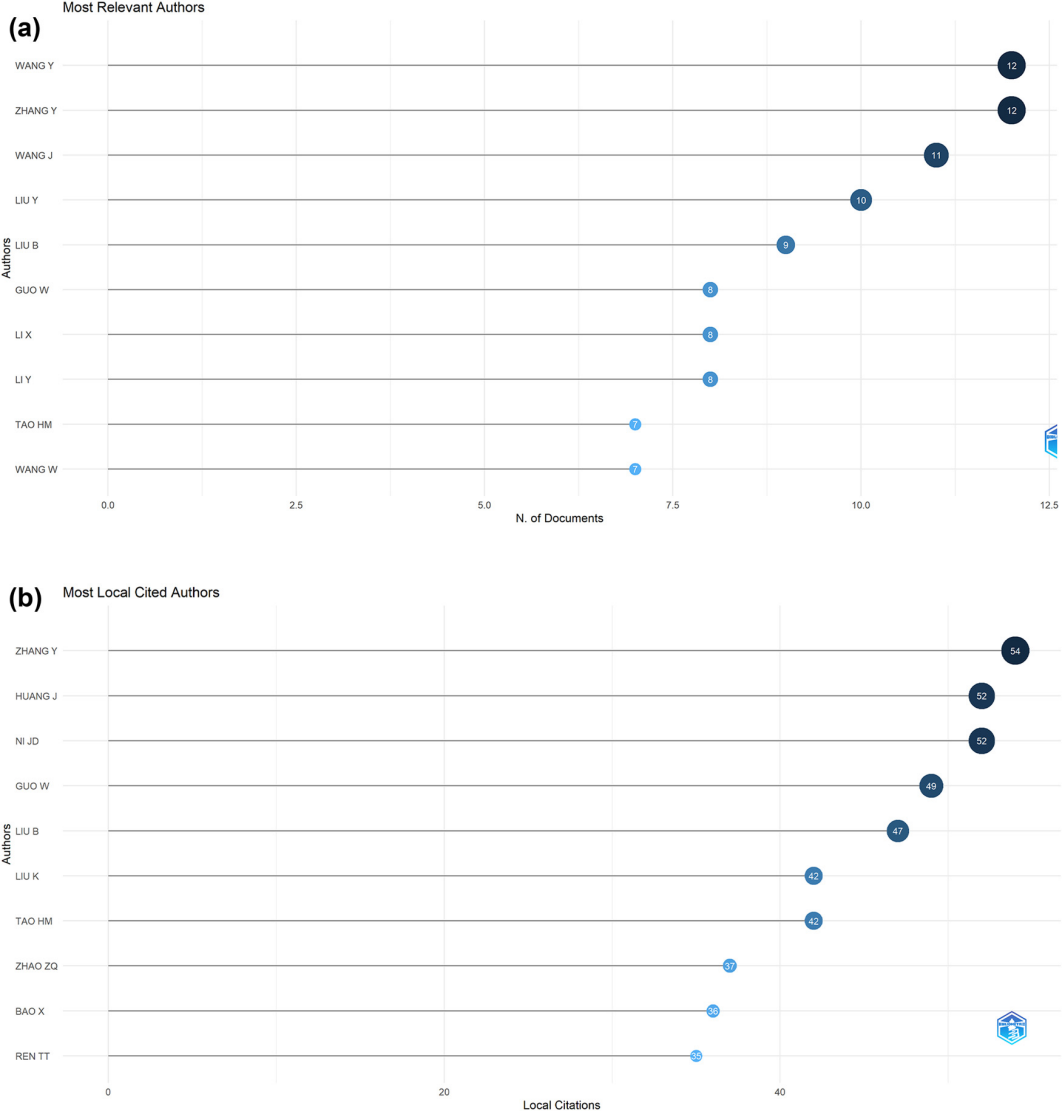
Visualization of the top 10 most relevant authors (a) and most local cited authors (b) in research on autophagy in osteosarcoma.

**Figure 7:**
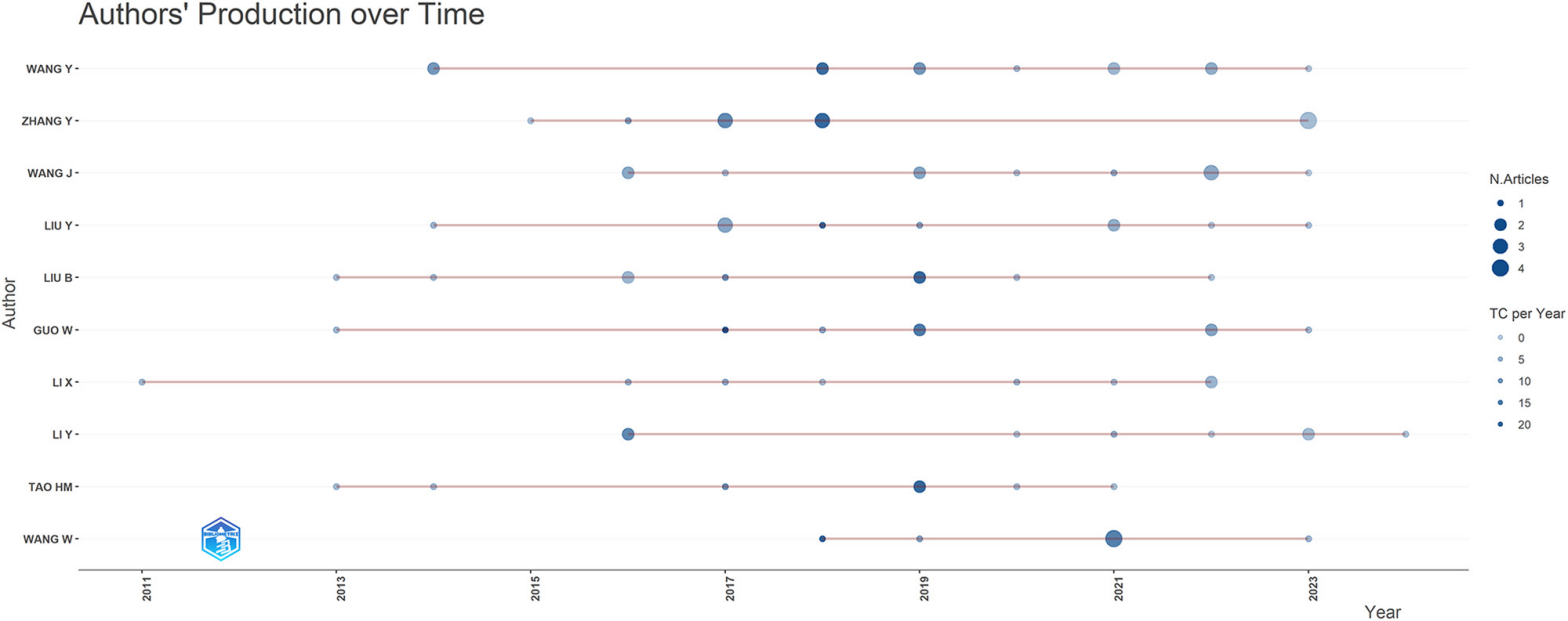
Visualization of the top 10 authors’ publications over time in research on autophagy in osteosarcoma.

Table 5 highlights the top 10 authors with the highest productivity in the field of autophagy and osteosarcoma research. Leading the list are Wang Yong from Inner Mongolia Medical University and Zhang Yuan from Chongqing Medical University, each with 12 publications. They are closely followed by Wang Jun from the Shanghai Key Laboratory of Orthopaedic Implants, who has contributed 11 publications, as illustrated in Figure 6a. Furthermore, Zhang Yuan’s publication volume has notably increased in recent years, as evidenced in Figure 7. Zhang Yuan has also accumulated the highest number of local citations, totaling 54, underscoring his significant influence and substantial contributions to this research area. Additionally, among the top 10 for local citations, scholars such as Huang Jun (*N* = 52), Ni Jiandong (*N* = 52), and Guo Wei (*N* = 49) stand out as prominent figures, as demonstrated in Figure 6b.

**Table 5:** The top 10 most relevant authors and most locally cited authors in the field of autophagy and osteosarcoma

Most relevant author	Count	Most locally cited author	Local citations
Wang Yong	12	Zhang Yun	54
Zhang Yuan	12	Huang Jun	52
Wang Jun	11	Ni Jiandong	52
Liu Ying	10	GuoWei	49
Liu Bin	9	Liu Bin	47
Guo Wei	8	Liu Ke	42
Li Xiaokang	8	Tao Huimin	42
LI Yi	8	Zhao Zhenqun	37
Tao Huimin	7	Bao Xing	36
Waang Wei	7	Ren Tingting	35

Significant collaboration among author clusters is also evident, as indicated by grouping authors who have published at least four articles, and distinguished by five different colors in Figure 5d. Based on the total link strength, Cai Zhengdong and Hua Yingqi, with values of 46 each, emerge as the most frequently collaborating authors.

### 3.5 Papers and co-cited references

**Figure 8:**
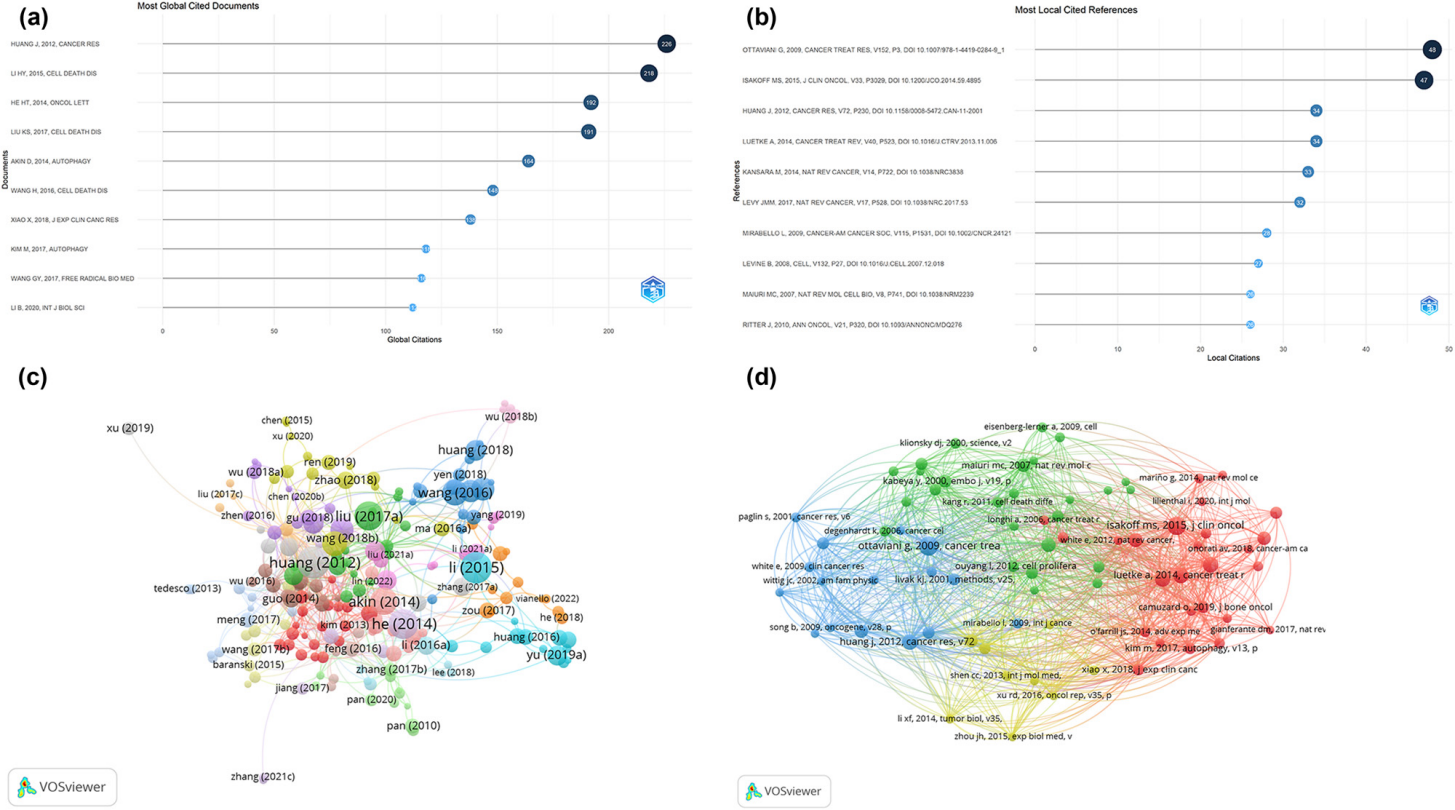
Visualization of the top 10 most globally cited documents (a), most locally cited references (b), cited papers (c), and co-cited references (d) in research on autophagy in osteosarcoma.

“Most Cited Papers” denote those with the highest citation counts within a specific database, subject area, journal, or timeframe, often signaling significant influence, innovation, or recognition in their fields. Citations act as a metric to assess a paper’s impact on the academic community. Identifying these papers highlights prevailing research trends, key issues, and substantial progress within a discipline. Of 395 papers, 257 received over 10 citations (Figure 8c). Table 6 lists the top 10 most cited papers, including four articles each with over 190 citations. The leading four, authored by Huang et al. [13] (226 citations), Li et al. [14] (218 citations), He et al. [15] (192 citations), and Liu et al. [16] (191 citations), demonstrate high academic recognition. The list is completed by papers from Akin et al. [17], Wang et al. [18], Xiao et al. [19], Kim et al. [20], Wang et al. [21], and Li et al. [22]. Remarkably, *Cell Death & Disease* and *Autophagy* journals each published multiple articles within the top 10, highlighting their role in disseminating pivotal research in this area.

**Table 6:** Top 10 most cited papers and most global cited documents

Title	DOI	First Author	Year	Journal	Citations
HMGB1 promotes drug resistance in osteosarcoma	10.1158/0008-5472.CAN-11-2001	Huang J	2012	Cancer Research	226
Celastrol induces apoptosis and autophagy via the ROS/JNK signaling pathway in human osteosarcoma cells: an *in vitro* and *in vivo* study	10.1038/cddis.2014.543	Li HY	2015	Cell Death & Disease	218
Molecular mechanisms of chemoresistance in osteosarcoma (Review)	10.3892/ol.2014.1935	He HT	2014	Oncology Letters	192
Apatinib promotes autophagy and apoptosis through VEGFR2/STAT3/BCL-2 signaling in osteosarcoma	10.1038/cddis.2017.422	Liu KS	2017	Cell Death & Disease	191
A novel ATG4B antagonist inhibits autophagy and has a negative impact on osteosarcoma tumors	10.4161/auto.32229	Akin D	2014	Autophagy	164
Erianin induces G2/M-phase arrest, apoptosis, and autophagy via the ROS/JNK signaling pathway in human osteosarcoma cells *in vitro* and in vivo	10.1038/cddis.2016.138	Wang H	2016	Cell Death & Disease	148
HSP90AA1-mediated autophagy promotes drug resistance in osteosarcoma	10.1186/s13046-018-0880-6	Xiao X	2018	Journal of Experimental & Clinical Cancer Research	138
GFRA1 promotes cisplatin-induced chemoresistance in osteosarcoma by inducing autophagy	10.1080/15548627.2016.1239676	Kim M	2017	Autophagy	118
Arsenic sulfide induces apoptosis and autophagy through the activation of ROS/JNK and suppression of Akt/mTOR signaling pathways in osteosarcoma	10.1016/j.freeradbiomed.2017.02.015	Wang GY	2017	Free Radical Biology and Medicine	116
Metformin induces cell cycle arrest, apoptosis, and autophagy through ROS/JNK signaling pathway in human osteosarcoma	10.7150/ijbs.33787	Li B	2020	International Journal of Biological Sciences	112

“Most Global Cited Documents” broaden the notion of “Most Cited Papers” to encompass papers that have garnered the highest citation counts worldwide across various databases and disciplines. This distinction highlights their far-reaching impact across numerous fields, marking substantial interdisciplinary achievements, theoretical advancements, or technological innovations. Such documents are celebrated for bridging gaps between disciplines and achieving broad recognition within the international scientific community. Intriguingly, the top 10 Most Global Cited Documents align with the top 10 most cited papers, as detailed in Table 6 and Figure 8a. Notably, “HMGB1 promotes drug resistance in osteosarcoma” [13] emerges as the preeminent paper globally, earning acclaim from the scientific community at large.

“Co-cited references” are defined as two or more scholarly works cited together within another research publication. This phenomenon indicates a thematic or methodological linkage, with frequent co-citation suggesting strong relevance or complementary findings among the works. Co-citations serve as a tool to uncover the interconnectedness across different research domains or to pinpoint foundational works within a particular field. Our co-citation analysis unveiled 14,734 references, with co-citations ranging from 1 to 48 (Figure 8d). In the realm of autophagy and osteosarcoma research, the most co-cited articles include Ottaviani and Jaffe [23] (48 citations), Isakoff et al. [24] (47 citations), Huang et al. [13], and Luetke et al. [25], each receiving 34 citations (Table 7). The subsequent highest-ranked articles received 26 and 33 citations, underscoring their significant influence in this research area.

**Table 7:** Top 10 most co-cited references and most locally cited references

Title	DOI	First author	Year	Journal	Citations
The epidemiology of osteosarcoma	10.1007/978-1-4419-0284-9_1	Ottaviani G	2009	*Cancer Treatment Reviews*	48
Osteosarcoma: Current Treatment and a Collaborative Pathway to Success	10.1200/JCO.2014.59.4895	Isakoff MS	2015	*Journal of Clinical Oncology*	47
HMGB1 promotes drug resistance in osteosarcoma	10.1158/0008-5472.CAN-11-2001	Huang J	2012	*Cancer Research*	34
Osteosarcoma treatment - where do we stand? A state of the art review	10.1016/J.CTRV.2013.11.006	Luetke A	2014	*Cancer Treatment Reviews*	34
Translational biology of osteosarcoma	10.1038/NRC3838	Kansara M	2014	*Nature Reviews Cancer*	33
Targeting autophagy in cancer	10.1038/NRC.2017.53	Levy JMM	2017	*Nature Reviews Cancer*	32
Osteosarcoma incidence and survival rates from 1973 to 2004: data from the Surveillance, Epidemiology, and End Results Program	10.1002/CNCR.24121	Mirabello L	2009	*Cancer*	28
Autophagy in the pathogenesis of disease	10.1016/J.CELL.2007.12.018	Levine B	2008	*Cell*	27
Self-eating and self-killing: crosstalk between autophagy and apoptosis	10.1038/NRM2239	Maiuri MC	2007	*Nature Reviews Molecular Cell Biology*	26
Osteosarcoma	10.1093/ANNONC/MDQ276	Ritter J	2010	*Annals of Oncology*	26

The top 10 locally cited references align perfectly with the top 10 co-cited references, highlighting a significant consensus in influential research within this field (Table 7 and Figure 8b). Notably, “HMGB1 promotes drug resistance in osteosarcoma” [13] by Huang et al., published in *Cancer Research* in 2012, holds the third position in both co-cited and locally cited rankings. This underscores its critical role and widespread recognition as a fundamental contribution to research on autophagy and osteosarcoma.

### 3.6 References with citation burstness

**Figure 9:**
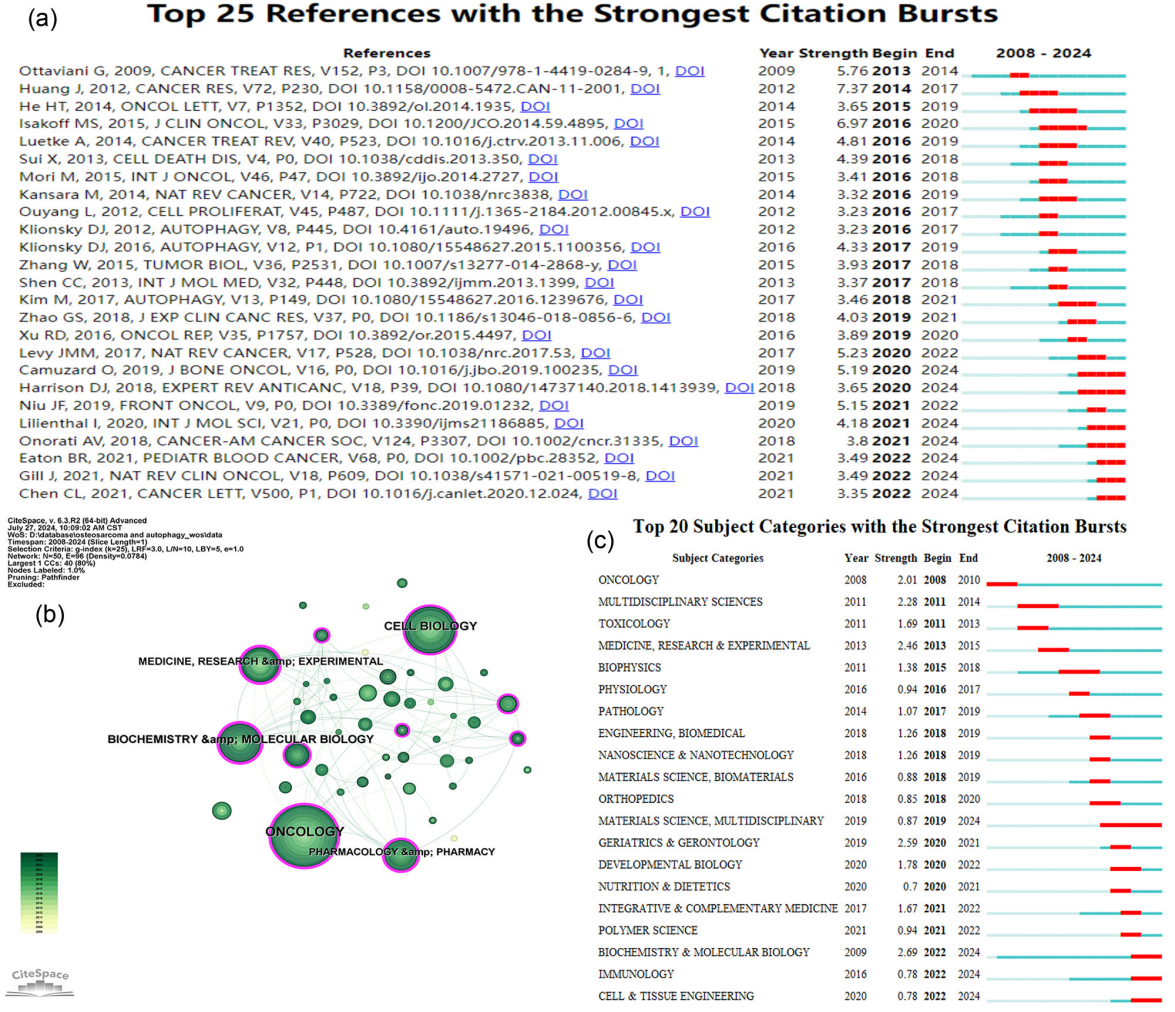
Visualization of the top 25 references with the strongest citation bursts (a), subject category co-occurrence network (b), and the top 20 subject categories with the strongest citation bursts (c) in research on autophagy in osteosarcoma.

Citation burstness refers to a significant increase in citations for a paper over a short period. By analyzing these bursts, research trends in a specific field can be anticipated [26]. The top 25 references with the strongest citation bursts were identified, with a minimum burst duration set to 2 years (Figure 9a). In the figure, the blue line denotes the year of the outbreak, while the red line indicates the period from the start to the end of the co-cited reference. “Strength” signifies the burst intensity; higher values indicate greater strength and influence of the publication [27].

Among the top 25 references with the strongest citation bursts, the one with the greatest burst strength was published by Huang et al. [13] in *Cancer Research* in 2012. This study demonstrates that the DNA-binding protein HMGB1 induces chemoresistance in osteosarcoma by promoting autophagy, offering a novel target for therapy improvement. Additionally, within these 25 references, the citation bursts of seven references ended in 2024, reflecting the latest research trends in autophagy and osteosarcoma research, and will be further discussed.

Among these seven references, the one with the highest burst strength was published in the *Journal of Bone Oncology* by Camuzard et al. [28]. This review summarizes the dual role of autophagy in osteosarcoma, highlighting its potential as both a pro- and anti-tumoral process and its implications for novel therapeutic targets. Lilienthal and Herold [29] published the study with the second-highest citation burst in the *International Journal of Molecular Sciences* in 2020. This review systematically introduces the molecular factors influencing treatment success and resistance in osteosarcoma, aiming to improve therapy by targeting resistance mechanisms and reducing toxicity.

The publication with the third highest citation burst was published by Onorati et al. [8] in *Cancer* in 2018. This review summarizes the dual role of autophagy in cancer, highlights the use of hydroxychloroquine in clinical trials, and discusses new autophagy inhibitors, suggesting autophagy as a promising target for cancer therapy. Harrison et al. [30] published the paper with the fourth-highest citation burst in *Expert Review of Anticancer Therapy* in 2018. The article reviews existing treatments and emerging strategies, emphasizing the need for novel research due to stagnant survival rates and highlighting ongoing clinical trials and innovative research on new agents and surgical techniques.

Additionally, Eaton et al. [31] published the paper “Osteosarcoma” in *Pediatric Blood & Cancer*, and Gill and Gorlick [32] published the paper “Advancing therapy for osteosarcoma” in *Nature Reviews Clinical Oncology*, both with the same citation burst intensity. The article “Osteosarcoma” reviews the multidisciplinary management of osteosarcoma, detailing standard radiotherapy guidelines in North America and Europe, and emphasizing the roles of chemotherapy, surgery, and radiotherapy in treatment. The article “Advancing therapy for osteosarcoma” discusses the potential for improved survival rates through molecular profiling, robust model systems, and targeted therapies, including antibody-drug conjugates and immune-checkpoint inhibitors, highlighting new therapeutic opportunities informed by recent biological insights.

Finally, Chen et al. [33] published the study with the seventh-highest citation burst in *Cancer Letters* in 2021. The study reviews recent advances in immunotherapy for osteosarcoma, discussing mechanisms, clinical trials, and future therapies, highlighting the potential for improved outcomes for patients with metastatic or recurrent osteosarcomas.

Through the analysis of these seven publications, one can find that the current research trends in the field of osteosarcoma focus on the roles of autophagy, molecular mechanisms of treatment resistance, innovative therapeutic strategies, multidisciplinary management, molecular profiling, and the potential of immunotherapy.

### 3.7 Analysis of subject categories

The knowledge map of the category co-occurrence network related to autophagy and osteosarcoma consists of 50 nodes and 96 links, as shown in Figure 9b. The top five subject categories by frequency of occurrence are *Oncology* (142), *Cell Biology* (82), *Biochemistry & Molecular Biology* [34], *Medicine, Research & Experimental* [35], and *Pharmacology & Pharmacy* [36] (Table 8). These categories represent the primary research areas in this field and have been extensively studied. The top five subject categories by betweenness centrality are *Biochemistry & Molecular Biology* (0.31), *Pharmacology & Pharmacy* (0.30), *Oncology* (0.22), *Medicine, Research & Experimental* (0.22), and *Cell Biology* (0.18) (Table 8). These fields are highly interconnected and serve as bridges in interdisciplinary research. The top 20 subject categories with the strongest citation bursts were identified with a minimum burst duration of 2 years (Figure 9c). Among these, the citation bursts of four categories ended in 2024. Thus, *Materials Science*, *Multidisciplinary, Biochemistry & Molecular Biology*, *Immunology*, and *Cell & Tissue Engineering* are currently hot research areas in this field.

**Table 8:** The top 10 frequency and centrality of subject categories related to autophagy and osteosarcoma

Rank	Subject categories	Frequency	Rank	Subject categories	Centrality
1	*Oncology*	142	1	*Biochemistry & Molecular Biology*	0.31
2	*Cell Biology*	82	2	*Pharmacology & Pharmacy*	0.30
3	*Biochemistry & Molecular Biology*	63	3	*Oncology*	0.22
4	*Medicine, Research & Experimental*	47	4	*Medicine, Research & Experimental*	0.22
5	*Pharmacology & Pharmacy*	38	5	*Cell Biology*	0.18
6	*Chemistry, Multidisciplinary*	19	6	*Biotechnology & Applied Microbiology*	0.16
7	*Multidisciplinary Sciences*	14	7	*Chemistry, Multidisciplinary*	0.14
8	*Biotechnology & Applied Microbiology*	13	8	*Chemistry, Applied*	0.14
9	*Biophysics*	11	9	*Toxicology*	0.13
10	*Biology*	11	10	*Nanoscience & Nanotechnology*	0.12

### 3.8 Keyword co-occurrence

#### 3.8.1 Keyword co-occurrence analysis

**Figure 10:**
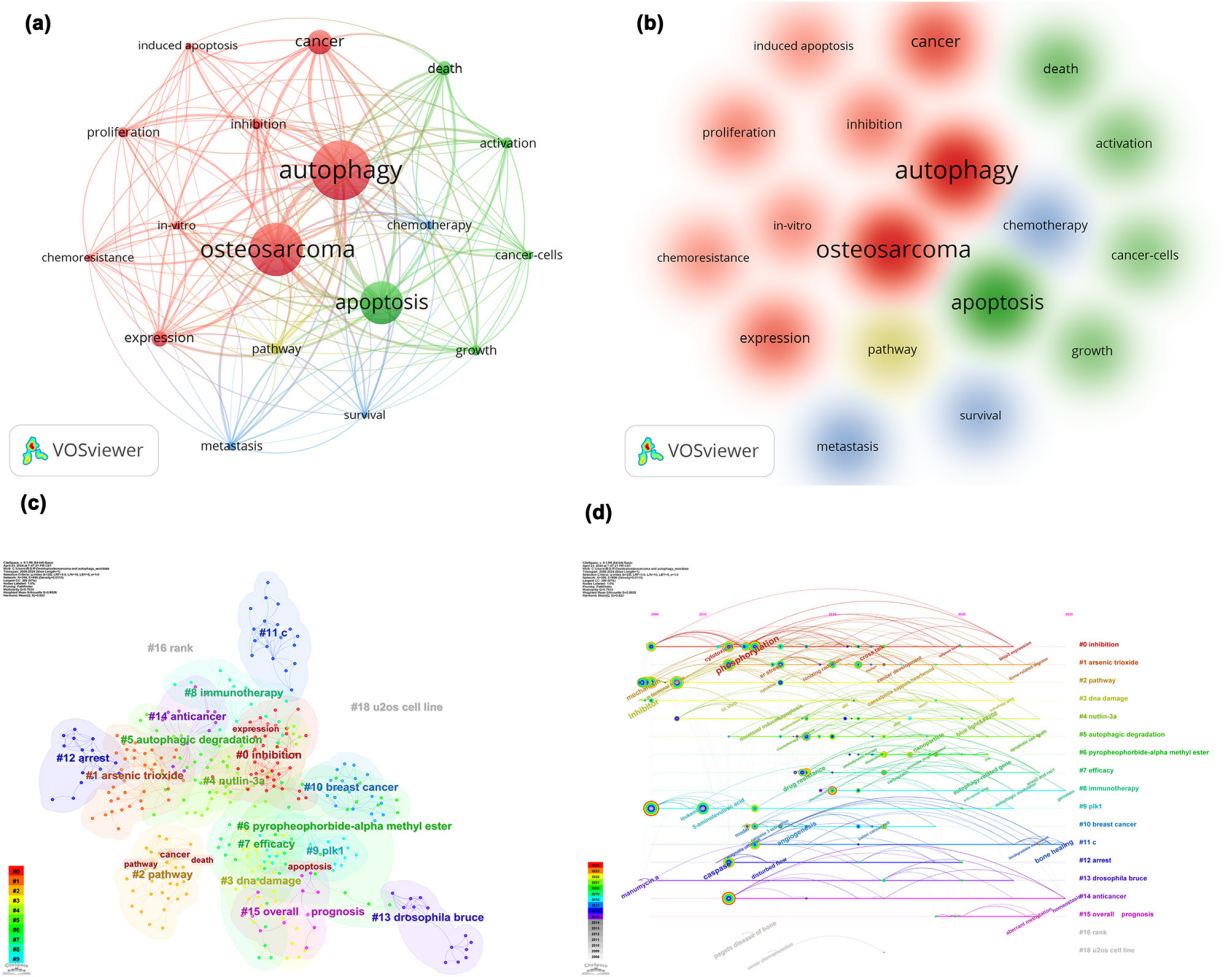
Visualization of the keyword co-occurrence network (a), keyword co-occurrence density (b), keyword clusters (c), and timeline graph (d) in research on autophagy in osteosarcoma.

From the co-occurrence analysis, 1,642 keywords were identified, elucidating research hotspots in autophagy and osteosarcoma. The most frequent keywords included “Autophagy” (266 co-occurrences) and “Osteosarcoma” (237), followed by “apoptosis” (191), “cancer” (109), “expression” [37], “death” [38], “inhibition” [39], “pathway” [39], “activation” [40], and “proliferation” [41]. To further illustrate other significant keywords in this field, “Autophagy” and “Osteosarcoma” were excluded from the subsequent analysis. Utilizing the remaining keywords, a network map was created, as depicted in Figure 10a and b. Among the keywords that appeared more than 20 times, 29 were highlighted and categorized into four thematic clusters: Group 1 (red) encompassed terms related to breast cancer, chemoresistance, and metastasis; Group 2 (green) focused on cellular mechanisms like autophagy and apoptosis; Group 3 (blue) discussed processes such as induction and inhibition; and Group 4 (yellow) addressed broader topics including cancer and therapy.

#### 3.8.2 Keywords cluster analysis

The analysis of autophagy in osteosarcoma can be effectively explored through network graph keyword clustering, which reveals hotspots and trends in this research area. The network graph comprises 399 nodes and 896 links, demonstrating a density of 0.0113. The keywords are clustered into 19 groups including “inhibition,” “arsenic trioxide,” “pathway,” “DNA damage,” “Nutlin-3a,” “autophagic degradation,” “pyropheophorbide-alpha methyl ester,” “efficacy,” “immunotherapy,” “PLK1,” and “breast cancer,” among others, as depicted in Figure 10c. The clustering quality, indicated by a *Q* value of 0.7633 and an *S* value of 0.8928, suggests a robust clustering configuration since both values exceed the thresholds of 0.3 and 0.5, respectively. The timeline graph indicates an increase in node activity starting from 2008, with most nodes concentrated between 2008 and 2019. The highest concentrations of citation outbreak nodes occur in group 0 “inhibition,” group 1 “arsenic trioxide,” and group 2 “pathway,” signifying key areas of research focus (Figure 10d). This research is primarily centered on the 19 themes identified.

These clusters can be categorized into five major research areas based on thematic and methodological consistency:

1. Therapeutic agents and their mechanisms include clusters such as “arsenic trioxide,” “Nutlin-3a,” and “pyropheophorbide-alpha methyl ester.” This area examines specific agents known to modulate autophagy in osteosarcoma cells. Research on arsenic trioxide, for instance, explores its role in promoting autophagic cell death in cancer cells [42]. Nutlin-3a, frequently studied for its p53-mediated anticancer effects, also impacts autophagic pathways [43]. Pyropheophorbide-a methyl ester is utilized in photodynamic therapy [44] and has been investigated for its efficacy in triggering autophagic cell death, presenting a novel therapeutic avenue.
2. Signaling pathways and molecular mechanisms consist of clusters like “pathway,” “DNA damage,” and “autophagic degradation.” This field investigates the molecular signaling pathways that regulate autophagy in osteosarcoma, focusing on key regulatory proteins and genes. Studies on DNA damage responses intersect with autophagy [45], examining how cells manage genotoxic stress, and exploring how osteosarcoma cells exploit autophagic degradation to maintain cellular homeostasis and respond to therapy [36].
3. Clinical strategies and treatment efficacy include clusters such as “inhibition [46],” “efficacy,” and “immunotherapy [33].” Research in this area assesses the effectiveness of autophagy-related treatments and their clinical applications in osteosarcoma [47], looking into the inhibition of autophagic processes as a therapeutic strategy, the overall efficacy of these interventions, and how modulating autophagy can enhance responses to immunotherapeutic agents [48].
4. Comparative and cross-cancer studies involve clusters like “breast cancer [49],” “PLK1,” and “U2OS cell line.” This domain comprises comparative studies that evaluate the role of autophagy in osteosarcoma relative to other cancers. For instance, research on PLK1, a kinase involved in cell cycle regulation [50], and the use of the U2OS osteosarcoma cell line help garner insights into autophagic regulation across different cancer types [51].
5. Prognostic factors and clinical outcomes are covered by clusters such as “anticancer” and “overall prognosis.” This area focuses on the prognostic significance of autophagy in osteosarcoma [41], exploring how autophagy-related factors affect patient outcomes and anticancer efficacy [52]. This research aims to correlate autophagic activity with clinical endpoints, supporting personalized medicine approaches.

#### 3.8.3 Keyword emergence analysis

**Figure 11:**
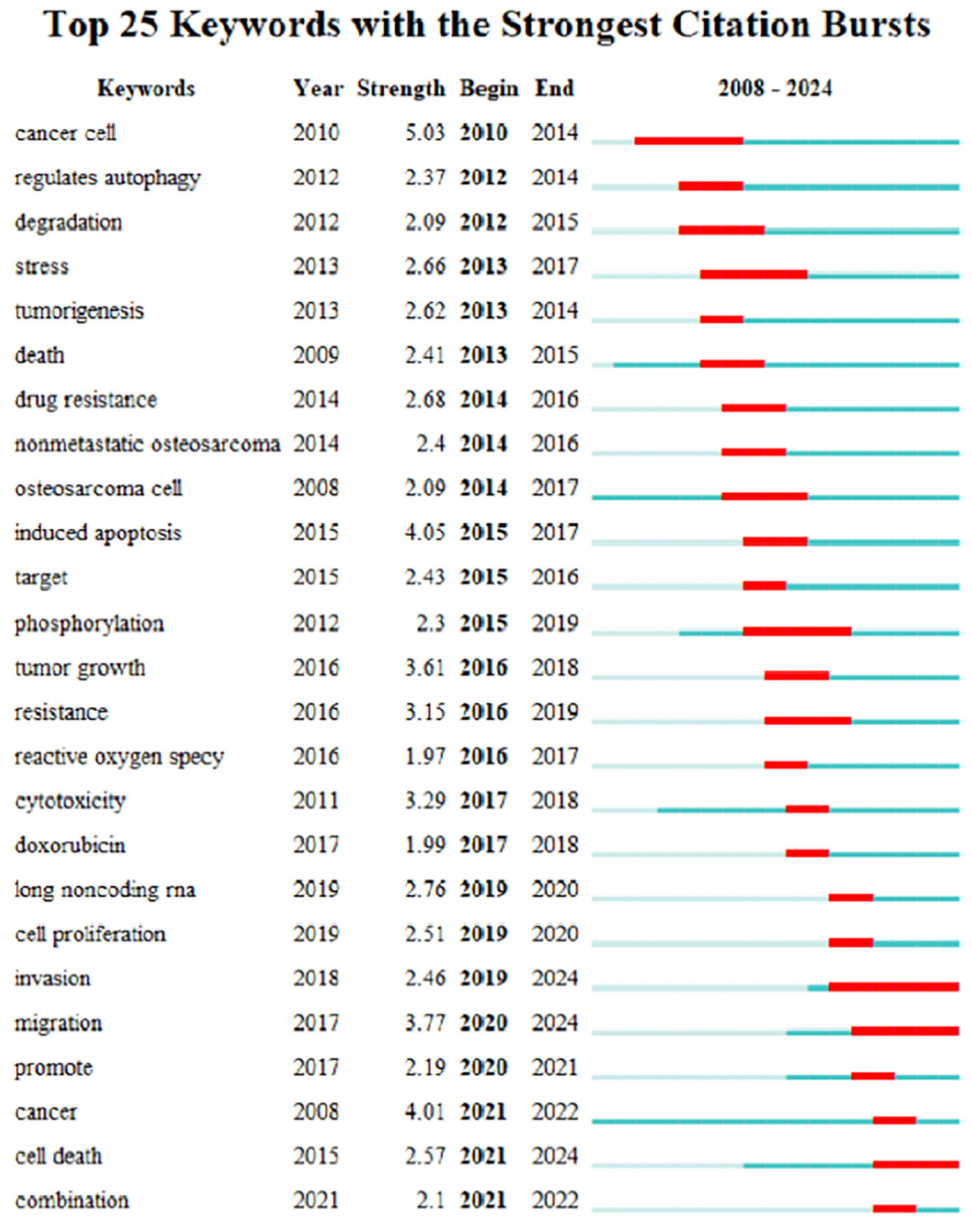
Visualization of the top 25 keywords with the strongest citation bursts in research on autophagy in osteosarcoma.

The analysis of keyword bursts is an emerging and vital approach for investigating the role of cellular autophagy in osteosarcoma. The keywords visualized in CiteSpace exhibited a significant rise in occurrences over a brief period, underlining the intensity and duration of these bursts, as illustrated in Figure 11. The study identified 25 keywords with bursts lasting over one year, each with a mean intensity value of at least 1.97. Notably, “cancer cell” displayed the highest burst intensity, with a value of 5.03, while “invasion” sustained the longest duration from 2019 to 2024. The persistence of keywords such as “invasion,” “migration,” and “cell death” suggests their potential to define future research trends. The keyword emergence chart categorizes these bursts into three phases: the initial phase focuses on exploring cellular mechanisms and therapeutic targets, the second phase examines clinical manifestations and responses to therapy, and the third phase is characterized by innovative treatments and integrated management strategies.

## 4 Discussion

### 4.1 General information

In the autophagy and osteosarcoma research domain, the initial publication emerged in 2008. Between 2008 and 2012, the field saw a modest output of five or fewer papers annually, suggesting its nascent phase. The publication volume modestly increased to between 13 and 30 articles yearly from 2013 to 2016, marking a period of gradual growth and exploration within the research community. A significant uptick occurred from 2017 to 2023, with the annual publication count reliably surpassing 30, indicating a burgeoning interest in this area of study. Remarkably, even before the completion of the first quarter of 2024, 10 papers have been published, continuing the upward trajectory of research output. This pattern reflects an escalating interest among scientists in understanding the implications of autophagy in osteosarcoma, pointing toward a thriving research landscape.

China leads globally in the number of publications within the autophagy and osteosarcoma research field, contributing 298 articles (75.44%), with the United States trailing with 43 articles (10.89%). Despite this, France outperforms in terms of average citations per article, boasting a count of 45.57. This disparity highlights that, although China is prolific in publication volume, its works garner fewer average citations compared to some other countries. In terms of international collaboration, co-authorship between the United States and China is notably high. At the institutional level, Shanghai Jiao Tong University leads with 31 publications, followed by Central South University with 9. However, publications from Central South University receive nearly double the average citations compared to those from Shanghai Jiao Tong University, suggesting a significant impact on the research community. The data suggest a correlation between citation counts and research influence, underscoring the value of deepened international cooperation to advance this scientific area further.

In the domain of autophagy and osteosarcoma research, *Cell Death & Disease* stands out as the most prolific journal, contributing 14 articles and comprising 3.54% of total publications – highlighting its prominence in the field. Among journals with substantial publication volumes, the *Journal of Experimental & Clinical Cancer Research* leads with the highest IF of 11.3, with *Cell Death & Disease* close behind at 9.0. Among the top 10 journals by publication volume, three are distinguished as Q1 (top quartile) journals. In terms of co-citations, three of the top 10 cited journals are ranked Q1, with two ranked Q2, underscoring that the most influential journals in autophagy and osteosarcoma research boast significant IFs.

In the domain of autophagy and osteosarcoma research, the most prominent authors, each with over 10 publications, include Zhang Yuan, Wang Yong, and Wang Jun. Zhang Yuan, affiliated with Chongqing Medicine University, leads with a total of 12 articles, establishing him as the field’s most influential researcher. His work reveals that TSSC3 promotes autophagy, effectively inhibiting the proliferation and dissemination of osteosarcoma. Furthermore, he suggests that the presence of TSSC3 in conjunction with ATG5 expression could potentially act as a promising prognostic marker for osteosarcoma patients [35]. Beyond his substantial publication record, Zhang Yuan also holds the distinction of being the most locally cited author in this area of study.

Considering the Most Cited Papers, Most Global Cited Papers, Most Co-Cited References, and Most Local Cited References, “HMGB1 Promotes Drug Resistance in Osteosarcoma” emerges as a seminal work in the discipline. It identifies HMGB1’s pivotal role in fostering chemoresistance in osteosarcoma by promoting autophagy, positioning it as an innovative target for enhancing treatment efficacy. This publication is foundational to the autophagy and osteosarcoma research domain, serving as a basis for subsequent investigations. Significantly, Zhang Yuan is acknowledged as the leading expert in this area, with “HMGB1 Promotes Drug Resistance in Osteosarcoma” [13] acclaimed as the most consequential paper.

### 4.2 Knowledge base

The co-citation analysis highlights key research areas in autophagy and osteosarcoma, with each referenced publication providing profound insights into the disease’s complex nature and therapeutic approaches. “The Epidemiology of Osteosarcoma” [23], distinguished as the foremost co-cited publication, has garnered 48 citations. This seminal study depicts osteosarcoma as the predominant bone cancer, noting its variable incidence across different age and demographic groups, particularly among adolescents and the elderly. It underscores the vital importance of comprehensive surgical excision for achieving optimal patient outcomes. Additionally, “Osteosarcoma: Current Treatment and a Collaborative Pathway to Success” [24] ranks as the second most-cited work, spotlighting chemotherapy advancements that have increased survival rates to between 65 and 70%.

Subsequent influential works include “HMGB1 Promotes Drug Resistance in Osteosarcoma” [13], the third most co-cited article, renowned for its extensive citations within this research domain. This research illuminates the role of the DNA-binding protein HMGB1 in imparting chemotherapy resistance in osteosarcoma, positioning it as a pivotal target for improving therapeutic results. Further investigations by Luetke et al. [25] and Kansara et al. [40] delve into systemic therapy enhancements for high-grade osteosarcoma and the promise of immunotherapy in addressing genomic challenges. Moreover, Levy et al.’s work [53] elucidates the dual role of autophagy in cancer progression, advocating for more comprehensive studies of its intricate effects as a route to innovative targeted therapies amidst ongoing discussions.

Mirabello et al.’s extensive analysis [50], ranked seventh in co-citations, meticulously explores the complexities of osteosarcoma across various demographics, emphasizing notable disparities in incidence, survival rates, pathological subtypes, and anatomical prevalence. This study highlights the disease’s diverse nature and its particular significance for individuals with Paget’s disease or secondary cancers. Other notable contributions by Levine et al. [54] and Maiuri et al. [55] examine autophagy’s role in health maintenance and its detailed interaction with apoptosis, respectively. Ritter and Bielack comprehensive [56] review promotes an integrated treatment strategy for osteosarcoma, stressing the crucial synergy between surgical and chemotherapeutic interventions. Among these foundational studies, “The Epidemiology of Osteosarcoma” and “HMGB1 Promotes Drug Resistance in Osteosarcoma” [13] are especially influential, with the latter recognized for its significant impact in both co-citations and total citations, marking its central role in autophagy and osteosarcoma research.

In summary, these works collectively indicate that current research predominantly aims to enhance treatment efficacy and elucidate the molecular mechanisms of the disease. Extensive investigations into drug resistance and the role of autophagy in disease progression are foundational for the development of targeted therapies with minimal side effects. Future research is poised to explore these molecular targets for precision medicine further. Considering the multifaceted role of autophagy at different cancer stages, its detailed functions at various disease stages warrant additional study. The successful application of immunotherapies in other cancers [57] also encourages further exploration in osteosarcoma treatment, especially to overcome genetic barriers and enhance survival rates. These insights equip future researchers to strategically select topics that address the ongoing challenges in this field.

### 4.3 Analysis of research hotspots

#### 4.3.1 Invasion

Osteosarcoma, a malignant tumor arising within the bones, most commonly impacts children and adolescents. It is marked by aggressive malignancy, characterized by swift growth and early metastasis, frequently spreading to the lungs and other bones [56]. Despite progress in medical treatments encompassing surgery, radiotherapy, and chemotherapy, osteosarcoma’s prognosis is often constrained by the timeliness and efficacy of diagnosis and treatment initiation [25]. Consequently, unraveling the pathogenesis of osteosarcoma is crucial for devising innovative treatment strategies.

Autophagy, the cellular process responsible for clearing damaged organelles and protein aggregates is pivotal in upholding intracellular homeostasis and mitigating undue cellular stress [3]. Emerging research delineates autophagy’s dualistic role in osteosarcoma’s progression [7]. It has been discovered that autophagy curtails osteosarcoma cell proliferation by promoting the removal of compromised mitochondria and protein clusters, thus obstructing the buildup of oxidative stress and DNA harm, which could forestall tumor advancement [5]. Experimental models have demonstrated a deceleration in osteosarcoma growth upon activation of autophagy through agents like rapamycin [6]. Conversely, autophagy empowers osteosarcoma cells to endure nutrient scarcity or exposure to chemotherapeutic agents. Notably, certain studies indicate that osteosarcoma cells diminish chemically induced apoptosis by augmenting autophagic activity in response to chemotherapy, implying that autophagy permits tumor cells to adapt to adverse conditions – thereby sustaining their viability, proliferation, and drug resistance, ultimately contributing to their aggressiveness [53].

Osteosarcoma’s invasiveness is evidenced by tumor cells’ capacity to infiltrate adjacent tissues and metastasize to remote sites. The complex role of autophagy in modulating osteosarcoma’s invasiveness is of notable importance. Research has demonstrated that suppression of autophagy-related genes, such as Beclin-1 and ATG5 [58], diminishes the migratory and invasive potential of tumor cells, attributed to reduced availability of energy and materials essential for these processes. This reduction highlights the possibility that intervening in autophagic pathways may decrease osteosarcoma’s invasiveness, presenting viable avenues for novel therapeutic interventions. On the contrary, other investigations reveal that autophagy activation in osteosarcoma cells facilitates environmental adaptation, thus augmenting their invasiveness and metastatic propensity. Enhanced autophagic flux enables these cells to more efficiently degrade and repurpose intracellular constituents, bolstering cell proliferation and spread under nutrient-deficient or hostile conditions. Navigating autophagy’s dual, opposing influences on osteosarcoma invasiveness is a prevailing challenge and focal point in current research endeavors.

In conclusion, osteosarcoma, a highly malignant tumor, is subject to the influences of numerous factors, including autophagy – a pivotal intracellular process exhibiting a dual role in the tumor’s progression. Investigating autophagy’s specific mechanisms within osteosarcoma promises to reveal novel therapeutic opportunities. There is a pressing need for future studies to deepen our understanding of the interplay between autophagy and osteosarcoma’s aggressiveness and to determine how autophagic modulation might enhance patient outcomes. Striking a therapeutic equilibrium, acknowledging autophagy’s facilitative and suppressive impacts, is imperative for advancing osteosarcoma treatment strategies.

#### 4.3.2 Migration

In confronting osteosarcoma, the cancer’s aggressive characteristics often impede effective treatment, leading to poor outcomes for patients. Nonetheless, the scientific community is relentlessly exploring novel therapeutic avenues, with autophagy targeting being recognized as a particularly promising approach [8]. The regulation of autophagy critically influences cancer cell survival, proliferation, and migration, positioning it as a key area of focus in the development of osteosarcoma treatments [3].

An intriguing research direction explores autophagy’s impact on osteosarcoma cells’ migration. Evidence suggests that autophagy’s effect on cancer cell mobility varies, influenced by the cellular environment and specific autophagic pathways engaged. For example, studies show that promoting autophagy in osteosarcoma cells diminishes their invasiveness, implying a defense against metastatic progression [59,60]. However, under certain circumstances, autophagy may be co-opted to enhance tumor dissemination [61], highlighting its multifaceted involvement in cancer development.

Research delving into autophagy’s dual role in osteosarcoma cell migration has aimed to clarify the specific pathways through which autophagy influences this process. Molecular studies have unveiled that genes and signaling pathways associated with autophagy are closely connected to cell motility mechanisms [62]. Such discoveries pave the way for targeted therapeutic strategies that leverage knowledge of autophagy’s particular mechanisms to inhibit osteosarcoma metastasis.

Furthermore, the dynamic relationship between autophagy and the tumor microenvironment adds layers of complexity to its influence on osteosarcoma development [3]. Environmental stressors within the tumor, like hypoxia and limited nutrient availability, can initiate autophagic processes that variably inhibit or facilitate cell motility. Grasping the nuances of these interactions is pivotal for crafting treatments capable of modulating autophagy to enhance therapeutic efficacy in osteosarcoma management.

As the search for innovative treatment approaches continues, both clinical and preclinical studies are progressively centering on the modulation of autophagy. Targeting this crucial cellular process, scientists are dedicated to crafting therapeutic interventions aimed at arresting osteosarcoma progression, thereby fostering optimism for enhanced patient prognoses. The active investigation into the role of autophagy within osteosarcoma highlights its significance as a therapeutic avenue, heralding potential breakthroughs in combating this challenging malignancy.

#### 4.3.3 Cell death

The pathophysiological underpinnings of osteosarcoma are complex, and characterized by a delicate equilibrium between cellular survival and death mechanisms, with autophagy playing an indispensable role [7]. Within the challenging milieu of osteosarcoma, characterized by metabolic stress and hypoxia, autophagy functions as a paradoxical force. While it supports cell survival through the recycling of compromised organelles and proteins, an overabundance of autophagic processes may precipitate autophagic cell death, or type II programmed cell death.

Recent studies have elucidated the complex relationship between autophagy and cell death mechanisms in osteosarcoma, uncovering promising therapeutic opportunities. Notably, research conducted by Zhao et al. and others has revealed that activating autophagy via specific pathways, including PI3K/Akt/mTOR, can curb osteosarcoma cell growth and trigger apoptosis, effectively reducing tumor proliferation [35]. On the flip side, autophagy inhibition under particular circumstances has been observed to augment chemotherapy’s effectiveness, indicating that autophagy may confer a survival benefit to osteosarcoma cells faced with therapeutic pressures.

Subsequent investigations into the molecular dynamics of autophagy within osteosarcoma have pinpointed critical regulatory elements, notably Beclin-1 and LC3 [63]. Their expression levels are intricately linked to both tumor advancement and patient outcome prognostication. Therapeutically targeting these pivotal autophagy-associated molecules presents an innovative strategy for osteosarcoma treatment, to adjust the autophagic pathway to leverage therapeutic gains.

Furthermore, the tumor microenvironment significantly influences autophagy and cell death mechanisms in osteosarcoma. Elements including cytokines, growth factors, and cellular stressors notably affect the autophagic response, thereby altering tumor cell behaviors such as migration, invasion, and therapeutic resistance. Elucidating these intricate interactions presents a valuable pathway for the creation of targeted therapies aimed at shifting the autophagic equilibrium in favor of tumor suppression.

Given these insights, the scope for autophagy-targeted therapeutic interventions in osteosarcoma is extensive. Clinical trials, alongside *in vitro* and *in vivo* studies, are progressively centering on the use of autophagy modulators [64]. These approaches, whether applied independently or synergistically with current treatments, aim to improve outcomes for osteosarcoma patients. Leveraging autophagy’s dual role in regulating cell death and survival offers a promising avenue for crafting novel osteosarcoma treatment strategies, holding the potential to significantly enhance patient prognosis in facing this formidable malignancy.

#### 4.3.4 Breast cancer

Autophagy plays a dual role in breast cancer [65], serving both as a tumor suppressor and a survival mechanism under stress. Abdullah et al. found that inhibiting autophagy in breast cancer cells increases apoptosis when used alongside chemotherapy [38], suggesting a therapeutic approach that utilizes the autophagic pathway. Similarly, the induction of autophagy in hypoxic regions of breast tumors was found to enhance survival and contribute to resistance against stress-inducing therapies [34]. These studies highlight autophagy’s complex role in both the progression [66] and treatment of breast cancer, emphasizing its potential as a biomarker and a therapeutic target.

In osteosarcoma, the role of autophagy is similarly complex yet distinctly different. Research reported that autophagy protects osteosarcoma cells by promoting drug resistance [67], especially against agents that induce apoptotic cell death. On the other hand, a study indicated that enhancing autophagy with drugs can selectively induce cell death in osteosarcoma, suggesting a potentially effective therapeutic approach [68]. These findings clarify how autophagy significantly influences cell survival and drug response dynamics in osteosarcoma.

Comparative analysis of autophagy in breast cancer and osteosarcoma provides profound insights into molecular mechanisms, signaling pathways, and genetic factors. Autophagy is controlled by complex signaling pathways that involve crucial autophagy-related genes (ATGs) [69] and regulatory proteins such as mTOR [70], BECN1 [71], and PI3K [72]. In breast cancer, the PI3K/Akt/mTOR pathway, commonly activated in tumor cells, generally suppresses autophagy, promoting survival and resistance to chemotherapy [73]. Conversely, alterations in gene expression such as BECN1 and mutations in p53 [74] change the role of autophagy in tumor suppression and resistance to therapy in osteosarcoma.

Moreover, the relationship between autophagy and apoptosis is a subject of great interest. In breast cancer, autophagy delays the onset of apoptosis by degrading damaged proteins and organelles, enhancing resistance to apoptosis-inducing drugs. In osteosarcoma, autophagy can provide cytoprotection but may also lead to autophagic cell death under certain conditions, a mechanism distinct from apoptosis but contributing to cell death.

The study of autophagy’s intricate role in cancer highlights its essential cellular functions in the progression and response to treatment of conditions like breast cancer and osteosarcoma. The distinct yet overlapping roles of autophagy in these cancers demonstrate the potential for developing targeted therapies that manipulate autophagic pathways, tailored to specific types of cancer. Understanding the molecular and genetic foundations of autophagy in various cancers allows researchers to create more effective therapeutic strategies to improve patient outcomes. Ongoing research into autophagy, including its regulatory mechanisms and its interactions with other cellular processes, continues to be an essential avenue in the search for more effective cancer treatments.

### 4.4 Analysis of research trends

Understanding the dynamic interactions between autophagy and osteosarcoma has been crucial in advancing therapeutic strategies for this aggressive cancer type. This analysis delineates the evolving research trends, divided into three key phases, each reflecting shifts in scientific inquiry and therapeutic methods.

Phase 1: cellular mechanisms and therapeutic targets (2010–2014)

This initial phase concentrated on the fundamental aspects of cancer cells and their microenvironments, emphasizing autophagy’s role in regulating degradation, stress responses, and cell survival within osteosarcoma [37]. Research during this period examined how autophagy influences tumorigenesis and cell death, shedding light on mechanisms of drug resistance in non-metastatic osteosarcoma. Notable keywords included “cancer cells,” “autophagy,” “degradation,” “stress,” “tumorigenesis,” and “death.”

Phase 2: clinical manifestations and treatment responses (2014–2017)

The subsequent phase focused on the interactions between osteosarcoma cells and the autophagic processes, investigating how autophagy modulates apoptosis through targeted therapies [75] and phosphorylation pathways. Studies highlighted the intricate dynamics within the tumor microenvironment, such as tumor growth, resistance mechanisms [76], and the cytotoxic effects of agents like doxorubicin [77]. Key terms during this phase were “nonmetastatic osteosarcoma,” “osteosarcoma cell,” “autophagy-induced apoptosis,” “target,” and “phosphorylation.”

Phase 3: therapeutic innovations and integrated management strategies (2017–2024)

The most recent phase concentrates on pioneering treatment methods and comprehensive management strategies [78], including exploring the role of autophagy in cell proliferation, invasion, and migration [61]. This research has increasingly focused on integrating therapies to enhance osteosarcoma treatment efficacy, examining ways to leverage autophagy in promoting cancer cell death and reducing tumor viability [29]. Important keywords for this phase are “autophagy,” “cell proliferation,” “invasion,” “migration,” “promotion,” “cancer,” and “cell death.”

In conclusion, the bibliometric analysis of autophagy in osteosarcoma illustrates a clear progression from understanding basic cellular interactions to applying this knowledge clinically to develop innovative treatment strategies. Each phase builds on the previous one, reflecting an evolving comprehension of the complex role autophagy plays in osteosarcoma progression and treatment. Future research should continue to delve into these interactions, focusing on translational methods that can translate laboratory insights into clinical applications, ultimately enhancing patient outcomes.

## 5 Limitations of this article

This study, focusing on the role of autophagy in osteosarcoma through a bibliometric analysis, possesses certain limitations that merit consideration. First, the analysis was restricted to publications retrieved exclusively from the Web of Science Core Collection. Consequently, relevant studies indexed in other substantial databases such as China National Knowledge Infrastructure (CNKI), PubMed, or Embase were not included. Due to the distinct characteristics of each database, combining data from multiple sources could present challenges, potentially skewing the comprehensiveness or bias of the findings [79]. Moreover, the Web of Science Core Collection is renowned for its representation of prestigious, high-impact academic journals, which implies that while the data is authoritative, it might not fully represent all existing literature on the topic.

Additionally, the use of CiteSpace software constrained the inclusion of articles published in English over the past 17 years, thus limiting the breadth of the literature review. Any potentially valuable studies published in other languages or before this period were excluded, which might omit significant trends or findings in the field. Furthermore, due to the time constraints associated with the completion of this paper, recent publications available after the literature search was concluded were not incorporated. This exclusion might prevent the analysis from reflecting the most current developments and discussions in the field of autophagy in osteosarcoma.

These limitations suggest that while the findings provide valuable insights, they should be interpreted with caution and understood as a representation based on a specific dataset and methodological approach rather than an exhaustive overview of the domain. Future research could benefit from a more inclusive approach that considers a wider array of databases, includes studies from a broader time range, and incorporates publications in multiple languages.

## 6 Conclusion

In conclusion, this bibliometric study has provided a visualization of research and analysis in the field of autophagy and osteosarcoma over the last 17 years, capturing a growing scholarly interest. Through detailed investigations into collaborations among countries, institutions, and authors, it is evident that strengthening these partnerships can significantly enhance the quality of research outputs and deepen investigations into osteosarcoma treatments.

Our in-depth discussions and analysis of keyword clustering revealed that current research hotspots include drug resistance mechanisms, therapeutic target development, the dual role of autophagy in cancer progression, and genomic influences on immunotherapy. Focused research on these hotspots is crucial for a more precise understanding of treatment strategies for osteosarcoma. Emergence analysis of keywords has pinpointed the relationships between autophagy-related cellular behaviors such as invasion, migration, and cell death, and their implications for cancer therapy, suggesting these as pivotal areas for future research.

Looking ahead, emerging trends are likely to concentrate on integrating novel therapeutic innovations and comprehensive management strategies. Strengthening research on the causative factors of osteosarcoma and its prognosis through autophagy could offer new therapeutic avenues and personalized treatment plans that could substantially impact the management of the disease.

This analysis not only furthers our understanding of the current landscape but also facilitates the transition of research findings toward clinical application, providing a valuable framework for scholars aiming to explore advanced studies in the treatment of osteosarcoma. Our findings underscore the critical role of autophagy in the progression and treatment of osteosarcoma, laying a foundation for future research that could transform therapeutic approaches in clinical settings.

## Supplementary Material

Supplementary Table
