# Supplementary material for: Trends and future directions of autophagy in osteosarcoma: A bibliometric analysis
Source: Open Med (Wars). 2024 Dec 3;19(1):20241080. doi: 10.1515/med-2024-1080 (PMC11627063; doi:10.1515/med-2024-1080)
Supplement: Supplementary Table [file med-2024-1080-sm.pdf]

Supplementary material

Table S1: Summary of Hotspots and Frontiers related research

Reference number	Author	Year	Journal	Title	Main point	DOI
45	Ritter J	2010	Ann Oncol	Osteosarcoma	Successful treatment of osteosarcoma requires collaboration among a multidisciplinary team at specialized centers, incorporating thorough surgical removal of tumors and multi-drug chemotherapy, with ongoing challenges in treating unresectable or recurrent cases	10.1093/annonc/mdq276
25	Luetke A	2014	Cancer Treat Rev	Osteosarcoma treatment - where do we stand? A state-of-the-art review	Systemic therapy has improved outcomes for high-grade osteosarcoma, achieving a 60-70% 5-year survival for localized disease, though challenges with metastatic recurrence and axial disease persist	10.1016/j.ctrv.2013.11.006
3	Ning B	2023	Cancer Med	Autophagy and its role in osteosarcoma	Despite considerable progress, treatment for Osteosarcoma (OS) — the predominant bone cancer in youngsters and adolescents — has reached a standstill in survival rates, urgently calling for new therapeutic research. This piece emphasises autophagy's vital role, a cell-recycling process, in OS, proposing genes and pathways linked to autophagy as prospective treatment targets	10.1002/cam4.5407
7	Nehme G	2020	Adv Exp Med Biol	Autophagy in osteosarcoma	Osteosarcoma management grapples with unyielding chemotherapy resistance and stagnant survival rates, underscoring the urgency of	10.1007/978-3-030-43085-6_11

(Continued)

Table S1: *Continued*

Reference number	Author	Year	Journal	Title	Main point	DOI
					deciphering autophagy's ambiguous impact on cancer cell destiny and the search for efficacious inhibitors	
5	Niu J	2019	Front Oncol	Insight Into the role of autophagy in osteosarcoma and its therapeutic implication	The critical need for better osteosarcoma treatments underscores the potential of autophagy as a dual-purpose therapeutic target	10.3389/fonc.2019.01232
6	Yu WX	2020	Eur Rev Med Pharmacol Sci	Effects of rapamycin on osteosarcoma cell proliferation and apoptosis by inducing autophagy	Rapamycin reduces viability, curbs proliferation, and boosts apoptosis in human osteosarcoma MG-63 cells by activating autophagy, an effect amplified by Beclin-1 plasmid transfection	10.26355/eurrev_202001_20076
42	Levy JMM	2017	Nat Rev Cancer	Targeting autophagy in cancer	Autophagy serves as a cellular cleanup process with dual roles in cancer, leading to debate over its therapeutic targeting. This review suggests progressing with autophagy-focused cancer treatments by grasping its nuanced roles and leveraging contemporary clinical trial methodologies	10.1038/nrc.2017.53
47	Oh JY	2019	Cancers (Basel)	Synergistic Autophagy effect of miR-212-3p in zoledronic acid-treated in vitro and orthotopic in vivo models and in patient-derived osteosarcoma cells	Zoledronic acid (ZOL) demonstrates potential in treating osteosarcoma (OS) by halting cell growth and triggering cell death via autophagy, significantly aided by increased miR-212-3p levels. This finding supports the idea of a combined ZOL and miR-212-3p treatment approach for advanced OS	10.3390/cancers11111812
8	Onorati AV	2018	Cancer	Targeting autophagy in cancer	This review by Onorati et al. (2018) synthesizes the dual role of autophagy in cancer, discussing its implications in both tumor suppression and	10.1002/cncr.31335

(Continued)

Table S1: *Continued*

Reference number	Author	Year	Journal	Title	Main point	DOI
					progression, and highlights emerging therapeutic strategies, including clinical trials using hydroxychloroquine, to inhibit autophagy as a potential treatment for advanced cancers	
49	Liu J	2020	Cancer Manag Res	Knockdown of CircCRIM1 inhibits HDAC4 to Impede osteosarcoma proliferation, migration, and invasion and facilitate autophagy by targeting miR-432-5p	This research uncovers that circCRIM1 is notably elevated in osteosarcoma (OS) and reducing its expression curtails tumor proliferation and spread by encouraging autophagy via the miR-432-5p/HDAC4 pathway, presenting novel avenues for OS therapy	10.2147/CMAR.S253130
48	Liu X	2023	Heliyon	Diallyl trisulfide inhibits osteosarcoma 143B cell migration, invasion and EMT by inducing autophagy	Diallyl trisulfide (DATS), a garlic-derived compound, effectively curbs osteosarcoma cell proliferation, movement, and epithelial-mesenchymal transition, and promotes apoptosis and autophagy by shutting down the EGFR/PI3K/AKT/mTOR signaling pathway, indicating its promise as a therapeutic option for osteosarcoma	10.1016/j.heliyon.2024.e26681
50	Xie C	2020	Cell Transplant	miR-19 Promotes Cell Proliferation, Invasion, Migration, and EMT by Inhibiting SPRED2-mediated Autophagy in Osteosarcoma Cells	Research shows miR-19 levels are up in osteosarcoma, suppressing SPRED2, a suppressor that encourages autophagy to fight tumor growth. The miR-19/SPRED2 pathway could be a novel target for osteosarcoma therapies, aiming to mitigate cancerous traits by boosting autophagy	10.1177/0963689720962460
51	Zhang J	2021	Front Cell Dev Biol	Autophagy-related genes and long noncoding RNAs signatures as predictive biomarkers for osteosarcoma survival	Research identifies autophagy-related long noncoding RNAs and genes as prognostic markers in osteosarcoma, linking autophagy to the	10.3389/fcell.2021.705291

(Continued)

Table S1: *Continued*

Reference number	Author	Year	Journal	Title	Main point	DOI
					disease's progression and drug resistance, offering new targets for treatment and diagnosis	
52	Zheng YD	2023	Cell Biol Int	Bufalin induces apoptosis and autophagy via the Ca2+/CaMKKβ/AMPK/Beclin1 signaling pathway in osteosarcoma cells	Bufalin triggers interaction between autophagy and apoptosis in osteosarcoma cells, leading to cell death via the Ca2+/calmodulin-dependent protein kinase β/AMPK/Beclin1 pathway to kickstart autophagy and a caspase-dependent method for apoptosis	10.1002/cbin.12021
53	Almansa-Gómez S	2023	Int J Mol Sci	Autophagy modulation as a potential therapeutic strategy in osteosarcoma: current insights and future perspectives	Studies indicate that controlling autophagy might present new approaches for treating osteosarcoma, potentially boosting the success of chemotherapy and targeting cancer stem cells to avert relapse, but additional research is essential to fully grasp its mechanisms and refine its use in therapy	10.3390/ijms241813827
54	Wu Q	2023	Cells	Autophagy and breast cancer: connected in growth, progression, and therapy	This review highlights the dual role of autophagy in breast cancer, facilitating both tumor adaptation and resistance to therapies, and discusses the potential of targeting autophagy to enhance the efficacy of current treatments	10.3390/cells12081156
55	Abdullah ML	2021	Int J Mol Sci	Eugenol-Induced Autophagy and Apoptosis in Breast Cancer Cells via PI3K/AKT/FOXO3a Pathway Inhibition	Eugenol, a natural compound from essential oils, shows potent anti-cancer properties against triple-negative and HER2-positive breast cancer by inhibiting cell proliferation, inducing apoptosis through the caspase pathway, and promoting autophagic cell death	10.3390/ijms22179243

(Continued)

Table S1: *Continued*

Reference number	Author	Year	Journal	Title	Main point	DOI
56	Notte A	2015	Int J Biochem Cell Biol	Taxol-induced unfolded protein response activation in breast cancer cells exposed to hypoxia: ATF4 activation regulates autophagy and inhibits apoptosis	This study reveals that in breast cancer cells exposed to taxol, ATF4 activation—not directly linked to the UPR—facilitates autophagy and adaptation to chemotherapy, suggesting its potential as a prognostic biomarker for treatment resistance in hypoxic tumors.	10.1016/j.biocel.2015.02.010
57	Liang G	2020	Mol Cancer	Autophagy-associated circRNA circCDYL augments autophagy and promotes breast cancer progression.	This study demonstrates that the autophagy-associated circRNA circCDYL promotes breast cancer progression through the miR-1275-ATG7/ULK1 axis, with elevated levels of circCDYL correlating with increased tumor burden, reduced survival, and poorer therapeutic response, positioning it as a potential prognostic marker and therapeutic target in breast cancer	10.1186/s12943-020-01152-2
58	Luo M	2023	J Transl Med	Cirsiliol induces autophagy and mitochondrial apoptosis through the AKT/FOXO1 axis and influences methotrexate resistance in osteosarcoma	Cirsiliol has been shown to inhibit osteosarcoma cell proliferation and induce apoptosis through targeting AKT phosphorylation and enhancing FOXO1 expression, suggesting its potential as an effective treatment for osteosarcoma	10.1186/s12967-023-04682-7
59	Ma K	2016	Oncol Rep	Cinobufagin induces autophagy-mediated cell death in human osteosarcoma U2OS cells through the ROS/JNK/p38 signaling pathway	This study confirms that cinobufagin triggers both apoptosis and autophagic cell death in U2OS cells through activation of the ROS/JNK/p-38 axis, with autophagy playing a crucial role in the antitumor effects of cinobufagin	10.3892/or.2016.4782
60	Cao Y	2019	Clin Chim Acta	Autophagy and its role in gastric cancer.	This review discusses the critical role of autophagy in gastric cancer, highlighting its regulation by specific genes and signaling pathways, its dual	10.1016/j.cca.2018.11.028

(Continued)

Table S1: *Continued*

Reference number	Author	Year	Journal	Title	Main point	DOI
					function in tumor suppression and promotion, and the therapeutic potential of autophagy modulators.	
61	Kim YC	2015	J Clin Invest	mTOR: a pharmacologic target for autophagy regulation	This review outlines the central role of mTOR, a serine/threonine kinase, in cellular metabolism, autophagy regulation, and disease treatment, emphasizing the clinical potential of mTOR inhibitors	10.1172/JCI73939
62	Hu F	2021	Nat Commun	IL-6 regulates autophagy and chemotherapy resistance by promoting BECN1 phosphorylation	This study reveals that IL-6 promotes autophagy and chemotherapy resistance in colorectal cancer through the IL-6/JAK2/BECN1 pathway, suggesting that targeting this pathway may offer a new therapeutic strategy for CRC	10.1038/s41467-021-23923-1
63	Li X	2020	Mol Cancer	Autophagy and autophagy-related proteins in cancer	Autophagy, a type II programmed cell death mechanism, involves complex roles with autophagy-related (ATG) proteins in cancer, exhibiting both tumor-suppressive and tumor-promoting effects depending on the cancer stage and context, suggesting its potential as a target for therapeutic intervention	10.1186/s12943-020-1138-4
64	Yang H	2022	Cell Death Dis	Histocompatibility Minor 13 (HM13), targeted by miR-760, exerts oncogenic role in breast cancer by suppressing autophagy and activating PI3K-AKT-mTOR pathway	This study reveals that high expression of Histocompatibility Minor 13 (HM13) in breast cancer correlates with poor prognosis and that its downregulation suppresses tumor proliferation and metastasis, activates autophagy via ER stress, and inhibits the PI3K-AKT-mTOR pathway, establishing HM13 as a potential therapeutic target	10.1038/s41419-022-05154-4

(Continued)

Table S1: *Continued*

Reference number	Author	Year	Journal	Title	Main point	DOI
					influenced by miR-760, a tumor-suppressor in breast cancer	
65	Pan Z	2021	Carbohydr Polym	Chitooligosaccharides inhibit tumor progression and induce autophagy through the activation of the p53/mTOR pathway in osteosarcoma	This study demonstrates that Chitooligosaccharide (COS), used as a drug carrier, exhibits significant anti-tumor effects on osteosarcoma by inhibiting cell growth and metastasis, inducing apoptosis and autophagy through the p53/mTOR signaling pathway, and enhancing chemotherapy sensitivity in vitro, positioning COS as a promising therapeutic candidate for osteosarcoma treatment	10.1016/j.carbpol.2020.117596
66	Usman RM	2021	Asia Pac J Clin Oncol	Role and mechanism of autophagy-regulating factors in tumorigenesis and drug resistance	This review highlights the complex role of autophagy in promoting cancer progression and drug resistance, regulated by over 30 genes that impact cell division and tumor microenvironment across various cancers	10.1111/ajco.13449
67	Kim HJ	2013	Int J Oncol	Cytoprotective role of autophagy during paclitaxel-induced apoptosis in Saos-2 osteosarcoma cells	This study demonstrates that low concentrations of paclitaxel (PCX) effectively induce apoptosis in osteosarcoma cells through a mitochondrial-mediated, caspase-dependent pathway, with autophagy playing a protective role that, when inhibited, further enhances apoptosis, suggesting a potential combined treatment strategy using PCX and autophagy inhibitors for more effective chemotherapy	10.3892/ijo.2013.1884
68	Ji Z	2023	MedComm	Targeting signaling pathways in osteosarcoma: Mechanisms and clinical studies	This review highlights the identification of molecular biomarkers and signaling pathways in osteosarcoma, emphasizing their potential to	10.1002/mco2.308

(Continued)

Table S1: *Continued*

Reference number	Author	Year	Journal	Title	Main point	DOI
					improve diagnosis, target therapy, and integrate treatment strategies	
69	Niu G	2021	Mater Sci Eng C Mater Biol Appl	Melatonin and doxorubicin co-delivered via a functionalized graphene-dendrimeric system enhances apoptosis of osteosarcoma cells	The study designed a functionalized graphene-dendrimeric system using Fe3O4 nanoparticles as a magnetic nanocarrier for the targeted co-delivery of doxorubicin and melatonin to enhance anticancer efficacy. The system demonstrated high encapsulation efficiency, biocompatibility, and effective cancer cell targeting, with significant synergy in antitumor effects due to the down-regulation of specific apoptosis inhibitors	10.1016/j.msec.2020.111554
70	Jafari F	2020	Ann Diagn Pathol	Osteosarcoma: A comprehensive review of management and treatment strategies	This article reviews the complex etiology, diagnosis, and current treatment methods for osteosarcoma, emphasizing the development and effectiveness of various immunotherapeutic approaches, including vaccines, cell-based therapies, cytokines, and monoclonal antibodies, to enhance treatment outcomes for this aggressive cancer	10.1016/j.anndiagpath.2020.151654
71	Lilienthal I	2020	Int J Mol Sci	Targeting Molecular Mechanisms Underlying Treatment Efficacy and Resistance in Osteosarcoma: A Review of Current and Future Strategies	This review explores the stagnation in osteosarcoma therapy improvement, attributing it to the tumor's heterogeneity and resistance to chemotherapy, and discusses current treatment strategies that focus on molecular determinants of susceptibility and resistance to enhance therapeutic outcomes	10.3390/ijms21186885
